# Comparison of Public Responses to Containment Measures During the Initial Outbreak and Resurgence of COVID-19 in China: Infodemiology Study

**DOI:** 10.2196/26518

**Published:** 2021-04-05

**Authors:** Xinyu Zhou, Yi Song, Hao Jiang, Qian Wang, Zhiqiang Qu, Xiaoyu Zhou, Mark Jit, Zhiyuan Hou, Leesa Lin

**Affiliations:** 1 School of Public Health Fudan University Shanghai China; 2 Department of Infectious Disease Epidemiology London School of Hygiene & Tropical Medicine London United Kingdom; 3 NHC Key Laboratory of Health Technology Assessment Fudan University Shanghai China

**Keywords:** COVID-19, engagement, latent Dirichlet allocation, public response, sentiment, social media, topic modeling

## Abstract

**Background:**

COVID-19 cases resurged worldwide in the second half of 2020. Not much is known about the changes in public responses to containment measures from the initial outbreak to resurgence. Monitoring public responses is crucial to inform policy measures to prepare for COVID-19 resurgence.

**Objective:**

This study aimed to assess and compare public responses to containment measures during the initial outbreak and resurgence of COVID-19 in China.

**Methods:**

We curated all COVID-19–related posts from Sina Weibo (China’s version of Twitter) during the initial outbreak and resurgence of COVID-19 in Beijing, China. With a Python script, we constructed subsets of Weibo posts focusing on 3 containment measures: lockdown, the test-trace-isolate strategy, and suspension of gatherings. The Baidu open-source sentiment analysis model and latent Dirichlet allocation topic modeling, a widely used machine learning algorithm, were used to assess public engagement, sentiments, and frequently discussed topics on each containment measure.

**Results:**

A total of 8,985,221 Weibo posts were curated. In China, the containment measures evolved from a complete lockdown for the general population during the initial outbreak to a more targeted response strategy for high-risk populations during COVID-19 resurgence. Between the initial outbreak and resurgence, the average daily proportion of Weibo posts with negative sentiments decreased from 57% to 47% for the lockdown, 56% to 51% for the test-trace-isolate strategy, and 55% to 48% for the suspension of gatherings. Among the top 3 frequently discussed topics on lockdown measures, discussions on containment measures accounted for approximately 32% in both periods, but those on the second-most frequently discussed topic shifted from the expression of negative emotions (11%) to its impacts on daily life or work (26%). The public expressed a high level of panic (21%) during the initial outbreak but almost no panic (1%) during resurgence. The more targeted test-trace-isolate measure received the most support (60%) among all 3 containment measures in the initial outbreak, and its support rate approached 90% during resurgence.

**Conclusions:**

Compared to the initial outbreak, the public expressed less engagement and less negative sentiments on containment measures and were more supportive toward containment measures during resurgence. Targeted test-trace-isolate strategies were more acceptable to the public. Our results indicate that when COVID-19 resurges, more targeted test-trace-isolate strategies for high-risk populations should be promoted to balance pandemic control and its impact on daily life and the economy.

## Introduction

In December 2019, COVID-19 emerged in Wuhan, and propagated rapidly across China and worldwide [1]. In response, many countries implemented stringent large-scale containment measures such as lockdowns, quarantines, and suspension of mass gatherings. Following these containment measures, the number of new COVID-19 cases decreased significantly from its peak in the first half of 2020 [2-6]. In China, the government adopted a rapid nationwide lockdown in late January 2020 during the Chinese New Year holiday, which included stay-at-home orders, transportation block, closure of shops and schools, suspension of gatherings, and suspension of work after the national Spring Festival holiday (the Chinese New Year), when most individuals in China would travel across cities or provinces for family gatherings. The lockdown measure aimed to prevent movement and mass gatherings in order to limit community transmission of COVID-19. As the then epidemic was under control, many provinces lifted containment measures from late February 2020 [7].

Although very few COVID-19 cases were thereafter reported in China [7], smaller-scale resurgences occurred. On June 11, 2020, a confirmed case, not linked to international travel, was reported in Beijing, ending the city’s almost 2-month span of zero incidence of local infections [8-10]. During the resurgence, Beijing adopted a more targeted response strategy instead of the city-level lockdown that was implemented during the initial outbreak. This targeted response strategy, namely “test-trace-isolate,” principally consisted of nucleic acid testing of individuals who had contact with known cases, tracing of close contacts of confirmed or suspected cases, and isolation of vendors and customers of Xinfadi Market (the epicenter of COVID-19 resurgence in Beijing). By rapidly identifying Xinfadi Market as the disease epicenter, most businesses and schools in Beijing were allowed to remain operational. Furthermore, instead of suspending all modes of transport, restrictions were promptly applied to passengers or goods entering or leaving Beijing, which prevented the virus from spreading outside Beijing. Moreover, the health code system was widely used to identify the exposure risk within the population [11]. One month later, no new cases were reported, with a total of 335 confirmed cases, and on July 19, 2020, Beijing lifted its response strategy. Worldwide, there has also been a resurgence of COVID-19 since August 2020, and evidence-based response strategies are thus needed to fully prepare for yet another resurgence [6,12-15].

The public response may change from that during the initial outbreak to resurgence and hence needs to be monitored. Previous studies have reported that the public frequently discussed the containment measures on social media, including lockdown, test-trace-isolate strategies, suspension of gatherings, personal protection, social distancing, travel restrictions, and workplace closures [16-18]. At the early stage of the COVID-19 pandemic, the public complied with containment measures in many countries, but their compliance gradually weakened as the pandemic progressed [19]. Studies also reported that both Weibo and Twitter users expressed more negative sentiments in the early stage of the COVID-19 pandemic [20-22]. In late 2020, many countries faced a COVID-19 resurgence and restarted containment measures. In India, Twitter users held a positive viewpoint to the second lockdown, but the majority held a negative opinion regarding the third lockdown [23]. In the Philippines, the negative sentiments increased owing to food shortage and helplessness during the lockdown [24]. Prolonged containment measures may lead to decreased risk perception, increased negative sentiments, and fatigue with sustaining containment measures [25-27]. Therefore, it is necessary to continuously monitor the public’s response to containment measures and design effective public communication strategies. Previous studies have reported that public risk perception and negative sentiments (such as depression, anxiety, and frustration) were highly correlated with the implementation of containment measures [16,26,28,29]. However, to our knowledge, none of these studies has assessed public responses to containment measures during COVID-19 resurgence and compared them across different stages of the pandemic.

With the protraction of pandemics, containment measures need to be adjusted accordingly, and it is imperative to gain timely feedback on containment measures from the public. Social media has been increasingly recognized as a platform for social surveillance [7,30]. Compared to surveys, social media not only allows for the monitoring of fluctuations in public sentiment and responses over a longer period but also is limited by a low recall bias. Using a machine learning approach based on social media data, this study aimed to assess and compare public responses to containment measures during the initial outbreak and resurgence of COVID-19 in China, including the level of public engagement, sentiments expressed, and frequently discussed topics. This study presents primary data on the evolution of public responses toward the progression of the COVID-19 pandemic, which would help inform adjustments in containment measures. Understanding the shifts of public responses could be essential for policymakers to prepare for future resurgence of COVID-19 globally.

## Methods

### Study Design

This is a comparative study based on social media data. With over 500,000,000 users, Sina Weibo (China’s version of Twitter) is the most influential social media platform in China [31]. Weibo allows users to share information and opinions in real time through posts and has been widely used to identify public concerns during the COVID-19 pandemic. We compared the public response to containment measures between the initial outbreak and resurgence on the basis of the following indicators: (1) the number of relevant Weibo posts, (2) the prevalence of negative sentiments in Weibo posts, and (3) the proportion of frequently discussed topics on Weibo. All data in this study are publicly available, and this study is exempt from ethical approval.

### Data Collection

Weibo posts that contain specific words can be retrieved through Sina Weibo’s keyword search function. We programmed a crawler in Python to curate publicly available Weibo posts through a keyword search. Since COVID-19 spread across China during the initial outbreak and only propagated in Beijing during resurgence, we retrieved all COVID-19–related Weibo posts in China for the initial outbreak and posts in Beijing for resurgence. For both surges of the pandemic, we curated Weibo posts from 1 week before the outbreak to the time when affected areas began to lift their responses (January 13 to February 28, 2020, for the initial outbreak and June 4 to July 20, 2020, for resurgence). In total, we curated 8,985,221 Weibo posts, and the data set is available on GitHub [32].

### Data Preprocessing

The initial pool of Weibo posts was preprocessed using a Python script to exclude duplicates, remove hashtags, links, uniform resource locators, and user handles from each post to clean the text [33,34]. We extracted the last user’s comment if it was a repost, and we excluded Weibo posts that were irrelevant to the public’s response by matching patterns with Python [35]. Finally, keyword matching was carried out from all COVID-19–related Weibo posts to extract Weibo posts targeting 3 primary containment measures: lockdown, the test-trace-isolate strategy, and suspension of gatherings (Table 1). A flowchart of data collection and preprocessing is shown in Multimedia Appendix 1.

**Table 1:** Keywords related to containment measures associated with the COVID-19 pandemic.

Containment measure	Keywords
Lockdown	“封城”(city lockdown), “封村”(village lockdown), “封路”(block road), “封闭”(close), “响应”(public health response), “停运”(stop traffic), “暂时关闭”(temporarily closed)
Test-trace-isolate strategy	“体温监测”/“测温”(body temperature monitoring), “检测”(test), “排查”(trace), “隔离”(isolate), “强制”(enforce/enforcement), “控制”(control)
Suspension of gatherings	“暂停”(suspension of gathering), “停止”(call off), “取消”(cancel), “推迟”(put off school opening or returning to work), “延期”(postpone), “延长”(prolong vacation)

### Data Analysis

We analyzed public engagement, sentiments, and frequently discussed topics related to each of the 3 containment measures and compared them between COVID-19 resurgence and the initial outbreak. Public engagement toward containment measures was assessed on the basis of the daily number of related Weibo posts, which was compared with the daily number of new COVID-19 cases locally reported by the National Health Commission of China and Beijing Municipal Health Commission. Public sentiment toward containment measures was analyzed using the Baidu open-source sentiment analysis application programming interface and measured from the proportion of Weibo posts with negative sentiments. Frequently discussed topics regarding containment measures on social media were identified through latent Dirichlet allocation (LDA) topic modeling combined with manual annotation. 

Topic modeling is an unsupervised machine learning technique that can automatically identify underlying topics or clusters by identifying groups of words that often co-occur in a textual data set (ie, Weibo posts) [9,17,36]. LDA is a widely used topic modeling algorithm to identify the most common topics across social media platforms [36,37]. In LDA, each document (ie, a Weibo post) is assumed to contain different topics, and each topic can be captured from a set of words. This helps map the given documents to the set of topics, such that the words in each document can be mostly captured by those topics. We applied LDA topic modeling by separating all documents into 30 machine-generated topics, and every Weibo post was assigned to a topic that it most likely belonged to according to the LDA model. LDA outputs provide keywords of the 30 LDA-generated topics for each containment measure during the initial outbreak and resurgence (Multimedia Appendix 2).

Since LDA is an unsupervised text classification algorithm based on the “bag-of-words model” [38], it may sometimes misclassify documents or misidentify topics [39-41]; therefore, it is important to manually assess representative documents (in our case, Weibo posts). In this study, LDA outputs was verified and improved by 2 independent researchers (YS and QW) to analyze key words generated by the LDA model and manually review sample posts for each topic. If the Weibo posts in one of the machine-generated 30 topics were found to contain several exclusive subtopics, that topic was manually reviewed. Finally, a random sample of Weibo posts (>10%) and their assigned topics were reviewed by 2 independent researchers (YS and QW) for quality control.

## Results

### Findings Overview

**Figure 1:**
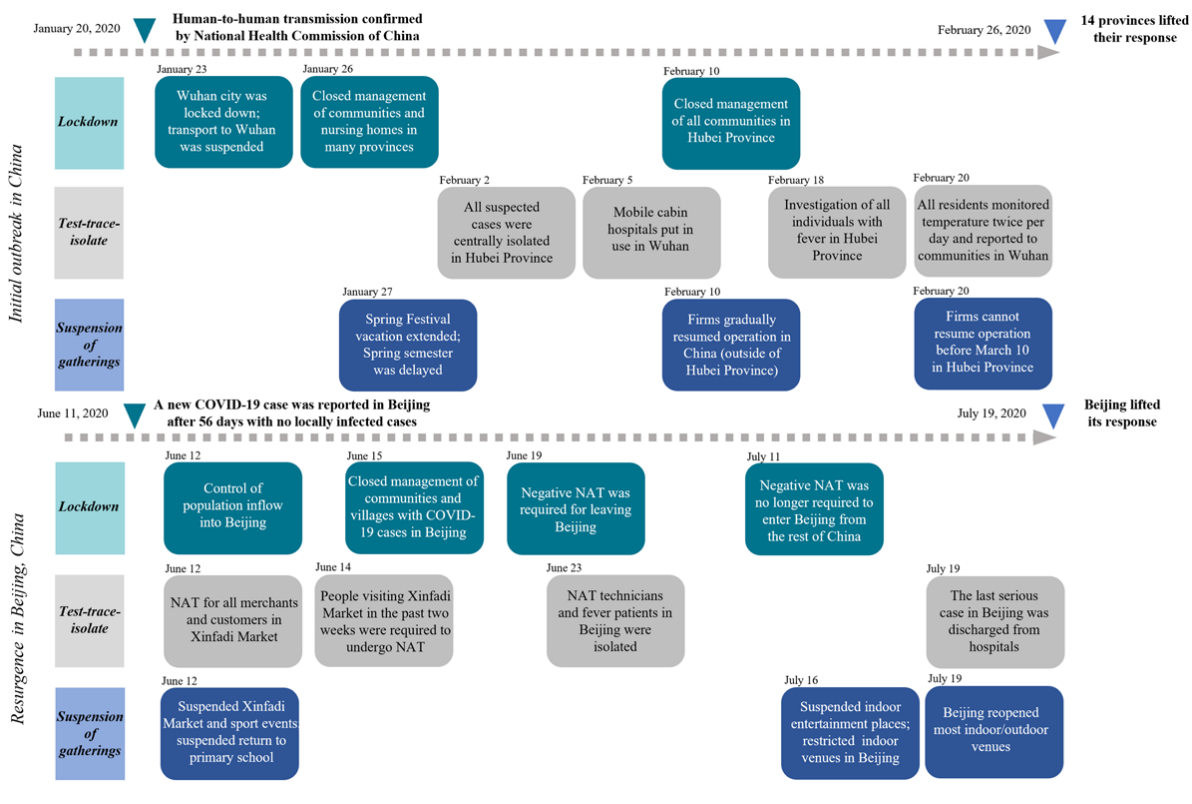
Timeline of containment measures during the initial outbreak and the resurgence of COVID-19 in China. Containment measures relevant to the lockdown, the test-trace-isolate measure, and suspension of gatherings are shown in chronological order. NAT: nucleic acid testing.

We compared the public sentiment to 3 containment measures during the initial COVID-19 outbreak in China and resurgence in Beijing (Figure 1): lockdown, the test-trace-isolate strategy, and suspension of gatherings. Generally, these measures evolved from a complete lockdown for the general population during the initial outbreak to a more targeted response strategy for high-risk populations during resurgence.

### Public Engagement With Containment Measures

**Figure 2:**
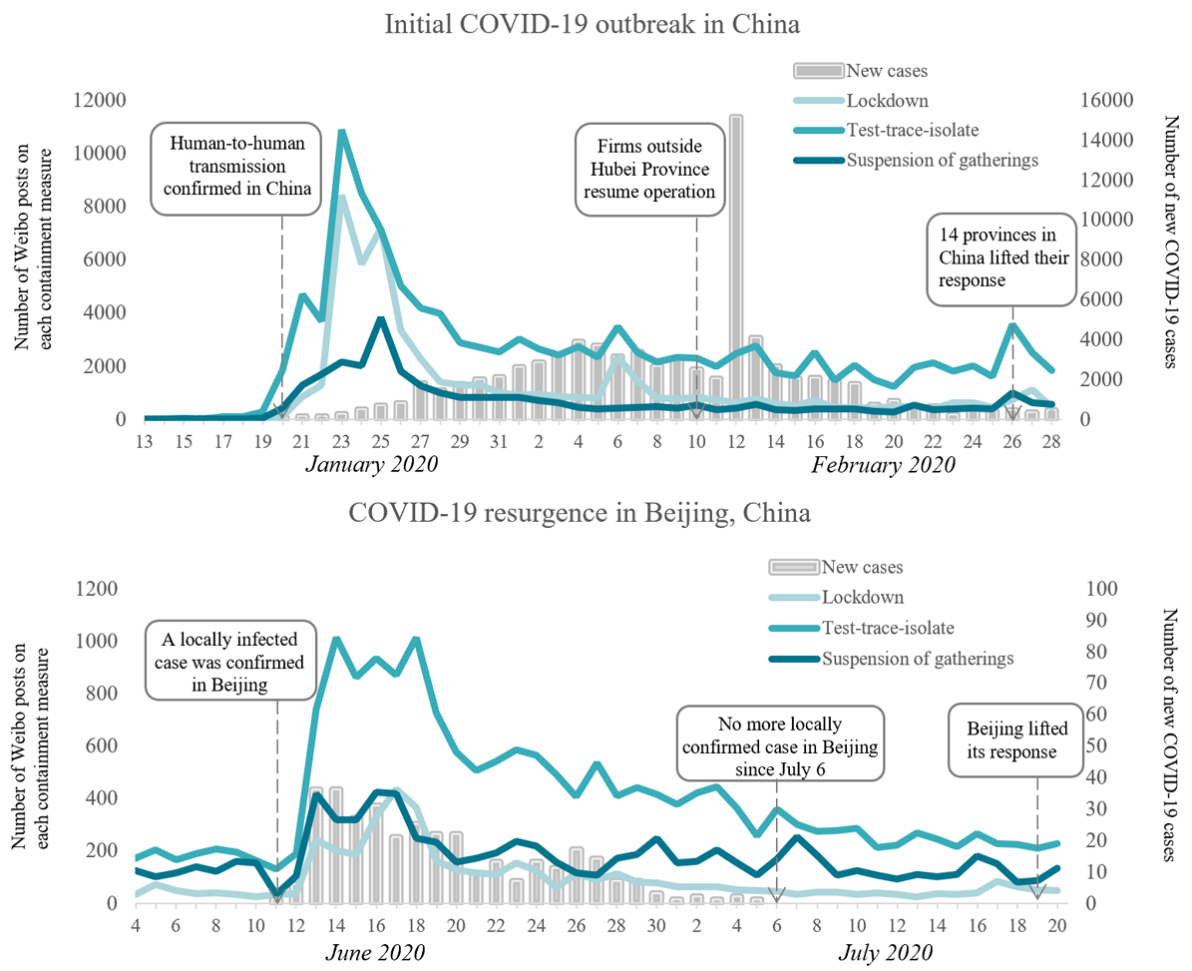
Number of Weibo posts on containment measures and new COVID-19 cases during the initial outbreak and the resurgence of COVID-19 in China. The lines show the daily number of Weibo posts on the lockdown, test-trace-isolate measure, and the suspension of gatherings. The bars show the daily number of new confirmed COVID-19 cases in China and Beijing.

The COVID-19 pandemic received marked engagement from the public nationwide. During both the initial outbreak and resurgence in China, test-trace-isolate measures had the most engagement from the public, and suspension of gatherings had relatively low engagement; one difference between these 2 periods is that lockdown measures had high engagement during the initial outbreak but the lowest engagement during resurgence (Figure 2). In both periods, the number of Weibo posts related to containment measures increased drastically after outbreak announcement and the implementation of containment measures, remained high for approximately 1-2 weeks, and then plummeted. From 1 week before to 1 week after the confirmation of human-to-human transmission (on January 20, 2020) in China, the average daily number of Weibo posts increased from 14 to 4168 for the lockdown measure, 69 to 2003 for the suspension of gatherings, and 340 to 6298 for the test-trace-isolate measure. Similarly, the average daily number of Weibo posts during the week before resurgence (on June 11, 2020) were 41 for the lockdown measure, 118 for the suspension of gatherings, and 179 for the test-trace-isolate measure. In comparison, this number approached 256 for the lockdown measure, 321 for the suspension of gatherings, and 803 for the test-trace-isolate measure in the week after resurgence. Furthermore, Weibo activity peaked approximately 1-2 weeks before the peak in COVID-19 cases, which indicated that Weibo seemed to track policy changes (eg, lockdown) rather than the actual number of cases.

### Public Sentiment Toward Containment Measures

**Figure 3:**
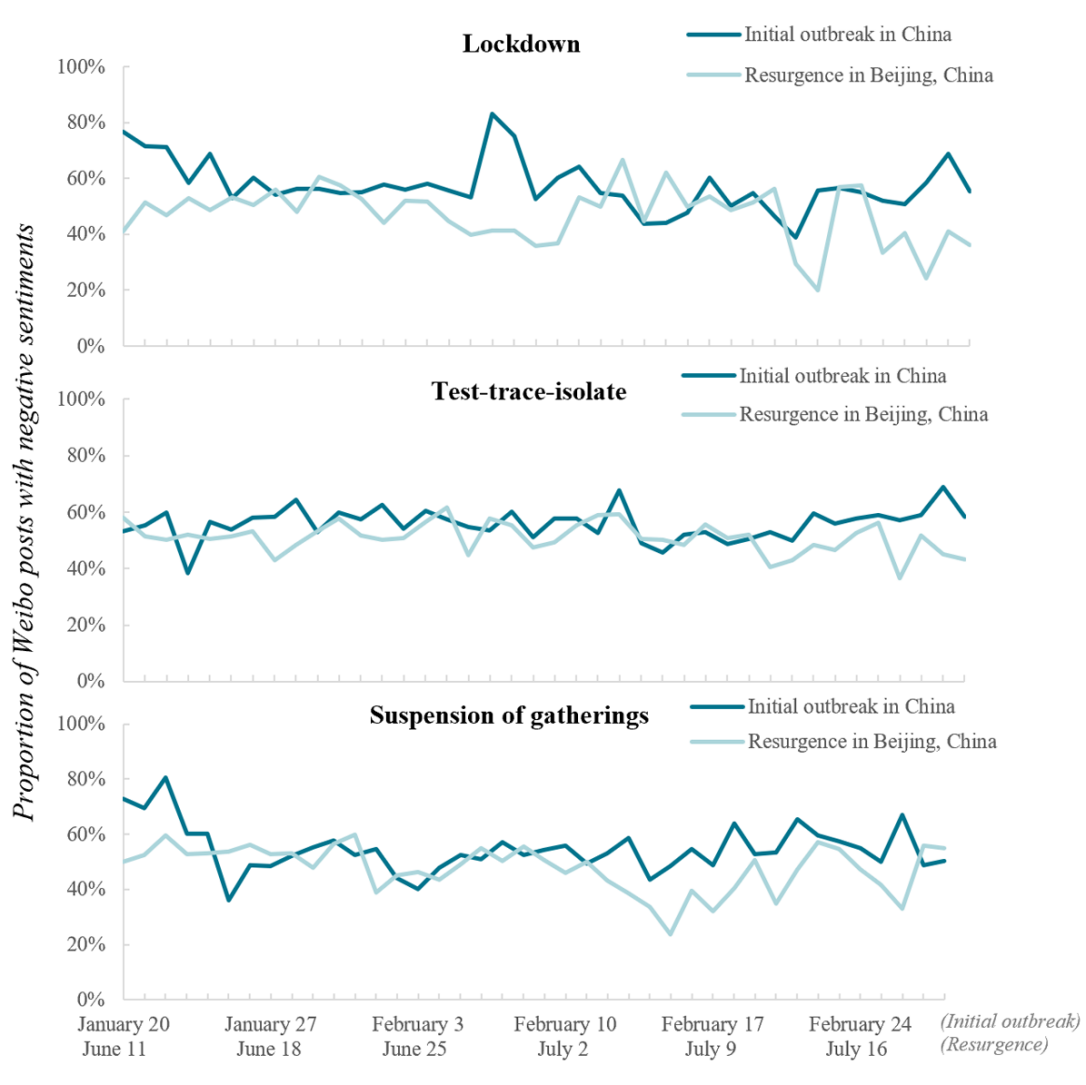
Proportion of Weibo posts with negative sentiments on containment measures during the initial outbreak and the resurgence of COVID-19 in China.

The overall negative sentiment toward all 3 containment measures during resurgence (average daily proportion of Weibo posts with a negative sentiment: 47% for the lockdown, 51% for the test-trace-isolate measure, and 48% for the suspension of gatherings) was lower than that during the initial outbreak (average daily proportion: 57% for the lockdown, 56% for the test-trace-isolate measure, and 55% for the suspension of gatherings) (Figure 3). During the initial outbreak, approximately 80% of the public immediately expressed negative sentiments toward the lockdown and suspension of gatherings after a lockdown was imposed in Wuhan, and approximately 60% of people expressed negative sentiments toward the test-trace-isolate measure. Thereafter, negative sentiments rapidly started to decrease for approximately 1 week and fluctuated surrounding a low level. However, for the test-trace-isolate measure, the proportion of negative sentiments varied through a smaller range than that for the other 2 containment measures. One exception is the spike in negative sentiments toward the lockdown on February 6, 2020, which might be related to concerns regarding the lack of medications or treatments for patients with other diseases; this accounted for a large proportion of posts expressing a negative sentiment on that day. During COVID-19 resurgence in Beijing, the negative sentiment toward containment measures was relatively lower and displayed lesser variation than that during the initial outbreak.

### Frequently Discussed Topics Related to Containment Measures

Weibo posts during the first two weeks of the initial outbreak and COVID-19 resurgence were used to identify frequently discussed topics on each containment measure.

#### Topics Related to the Lockdown Measure

**Figure 4:**
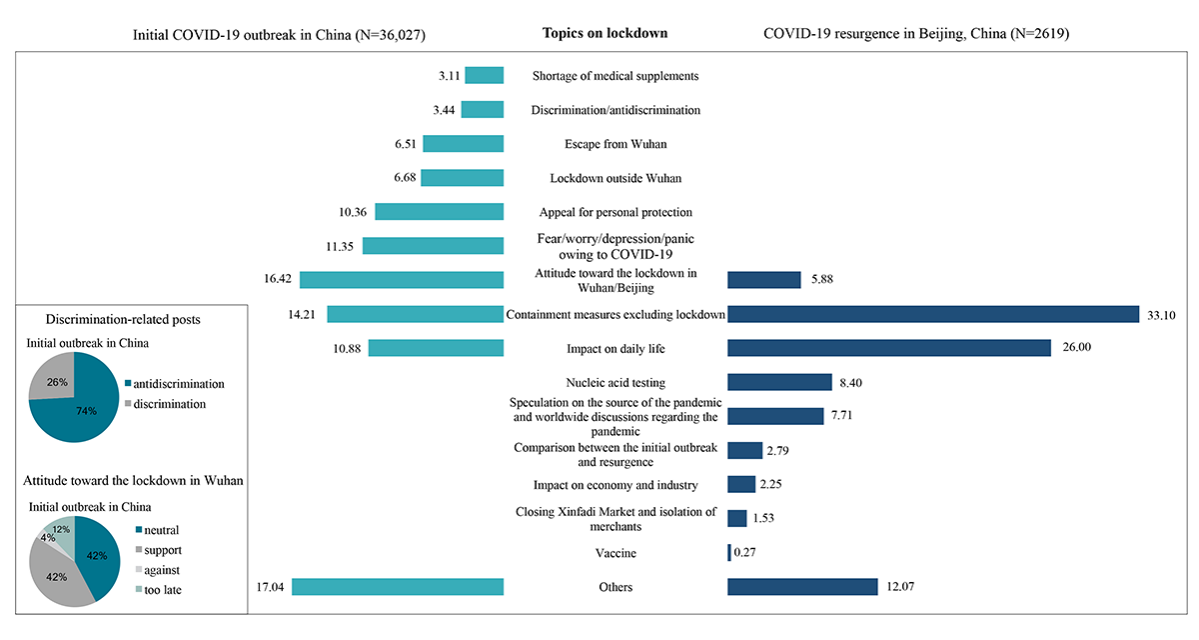
Proportion of posts related to frequently discussed topics on the lockdown measure during the initial outbreak and the resurgence of COVID-19 in China. The columns show the proportion of posts on each topic among all posts related to the lockdown measure. Pie charts show the proportion of various attitudes toward some topics during the initial outbreak.

For the initial outbreak, the top 3 topics related to the lockdown measure were attitudes towards the lockdown in Wuhan (n=5915/36,027, 16.42%), discussions on containment measures other than the lockdown (n=5120/36,027, 14.21%), and expressions of negative sentiments owing to the pandemic, such as fear, worry, depression, and panic (n=4090/36,027, 11.35%) (Figure 4). Among posts related to attitudes toward the lockdown in Wuhan, 2473 of 5915 (42%) posts expressed support for the policy, while 220 (4%) expressed opposition toward the policy, along with 720 (12%) posts that indicated that the lockdown should have been implemented earlier. Regarding resurgence in Beijing, the leading 3 topics were public containment measures (n=867/2619, 33.10%), impacts on daily life (n=681/2619, n=26.00%), and nucleic acid tests for COVID-19 (n=220/2619, 8.40%). Since more targeted responses had been used during the resurgence, containment measures excluding lockdown became the main focus of Weibo posts, whereas posts related to the lockdown decreased from 16.42% to 5.88%. After the resumption of work, people paid more attention to how containment measures would affect their daily lives or work, and thus the proportion of relevant posts increased from 10.88% to 26.00%. Consequently, there were discussions about the impact of containment measures on the economy and industry. Noticeably, people expressed more biases by ascribing the emergence and spread of the pandemic to Wuhan residents during the initial outbreak; in contrast, this was barely mentioned during resurgence in Beijing. Other topics during the initial outbreak included an appeal for personal protection (10.36%), lockdowns outside Wuhan (6.68%), people leaving Wuhan (6.51%); shortages in the supply of medical equipment, including masks, were not widely discussed (3.11%). Topics such as vaccines and nucleic acid tests only emerged during resurgence.

#### Topics Related to the Test-Trace-Isolate Measure

**Figure 5:**
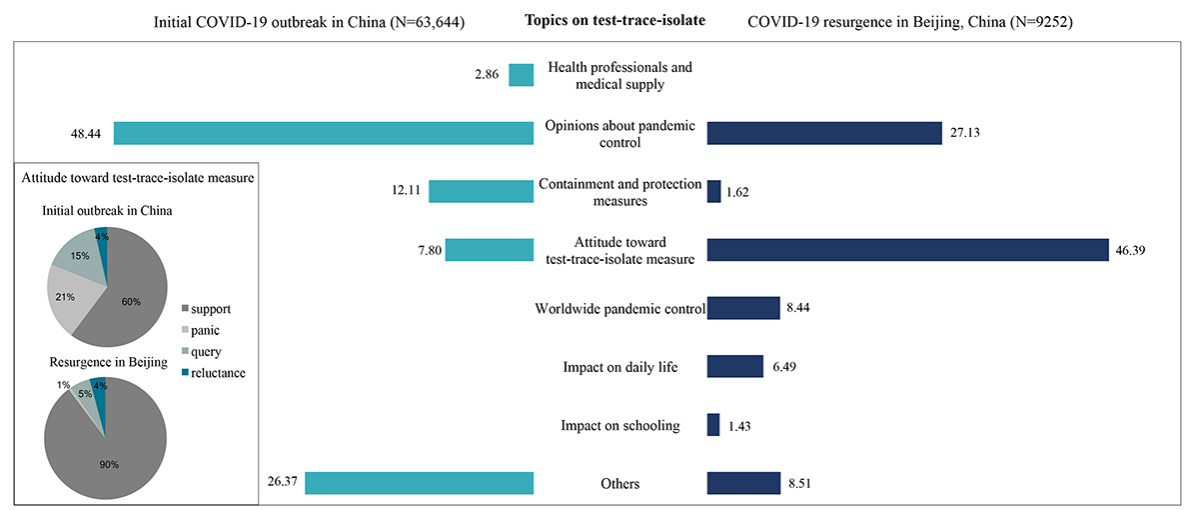
Proportion of posts related to frequently discussed topics on the test-trace-isolate measure during the initial outbreak and the resurgence of COVID-19 in China. The columns show the proportion of posts on each topic among all posts related to the test-trace-isolate measure. Pie charts show the proportion of various attitudes toward the test-trace-isolate measure between the initial outbreak and resurgence.

During the initial outbreak, 30,826 of 63,644 (48.44%) posts related to the test-trace-isolate measure were opinions on pandemic control, which expressed hope or trust in rapid control of the pandemic and contained suggestions about specific containment measures and descriptions of the status of its spread (Figure 5). During resurgence, the percentage of posts expressing opinions on pandemic control decreased to 27.13% owing to public confidence in effective response measures. The second-most popular topic during the initial outbreak was quarantine and protective measures, which were only indicated in 1.62% of posts during resurgence. In the initial outbreak, 7.8% of posts expressed public attitudes toward the test-trace-isolate measure, while during resurgence, 46.39% of users expressed their attitudes. This further illustrates how the discussion’s focus shifted from the status of pandemic control to targeted response measures. Interestingly, public attitudes toward the test-trace-isolate measure differed between these 2 periods of the pandemic in China. In the initial nationwide outbreak, only 3921 of 6506 (60%) posts supported the test-trace-isolate strategy. However, during resurgence in Beijing, the proportion of posts supporting the test-trace-isolate policies increased to 90% (n=3853/4292). Posts indicating panic decreased from 21% to 1%, and those related to queries decreased from 15% to 5%. Users expressing reluctance to be tested or quarantined accounted for 4% of posts during these 2 periods. Furthermore, different topics emerged during these 2 periods. During the initial outbreak, some posts (2.86%) discussed medical equipment and health professionals, while during resurgence, people discussed the impact of the test-trace-isolate measure on schooling, work, daily lives, and the status of the pandemic worldwide.

#### Topics Related to the Suspension of Gatherings

**Figure 6:**
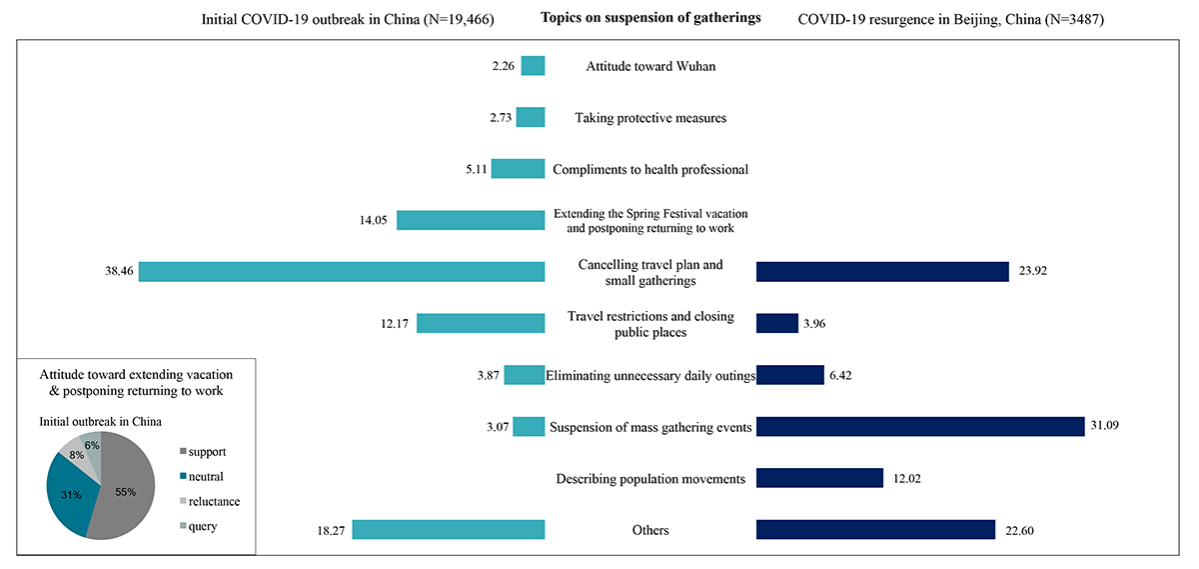
Proportion of posts related to frequently discussed topics on the suspension of gatherings during the initial outbreak and the resurgence of COVID-19 in China. The columns show the proportion of posts on each topic among all posts related to the suspension of gatherings. Pie charts show the proportion of various attitudes toward some topics during the initial outbreak.

Among posts on the suspension of gatherings, 7487 of 19,466 (38.46%) and 834 of 3487 (23.92%) posts indicated the cancellation of travel plans and small-scale gatherings during the initial outbreak and resurgence, respectively (Figure 6). The proportion of posts describing the suspension of mass gathering events increased drastically from 3.07% during the initial outbreak to 31.09% during resurgence, which indicates that the public response was much more sensitive to the suspension of mass gathering events such as sports and concerts, during resurgence. Other common topics during these 2 periods included travel restrictions, closure of public places, and elimination of unnecessary daily outings. In addition, 2735 of 19,466 (14.05%) posts discussed the extension of the Spring Festival holiday and postponing of the return to work during the initial outbreak, among which, 55% of users supported the extension of the holiday. During resurgence, discussions on issues regarding population movement emerged, such as visas and immigration.

## Discussion

### Principal Findings

Through social media surveillance, we analyzed and compared the level of public engagement, sentiment expressed, and frequently discussed topics related to 3 primary containment measures during the initial outbreak and the resurgence of COVID-19 in China. As the pandemic control strategy switched from lockdown to a targeted response during resurgence, public responses became more rational. During the initial outbreak, the number of Weibo posts escalated drastically after the lockdown in Wuhan, a high proportion of posts indicated a negative public sentiment toward all 3 primary containment measures, and topics related to discrimination emerged. During resurgence, however, the public showed less engagement and less negative sentiments, were more supportive towards containment measures, and shifted their focus to the impacts of containment measures on their daily lives or their work.

Monitoring of public responses for pandemic control strategies is crucial for obtaining rapid feedback and informing strategy adjustments during a pandemic. Previous studies have reported that social mobilization and community engagement were central to the Ebola response in West Africa [42-44]. During the COVID-19 pandemic, especially during the initial outbreak, Weibo users in China raised questions regarding the pandemic’s source, shortage of medical equipment, and discrimination against Wuhan residents. A considerable number of Weibo posts expressed panic or queries regarding containment measures. This is worth noting, since previous studies have reported that panic and queries regarding containment measures and discrimination may inhibit community engagement and dampen pandemic control [19]. During resurgence, public concerns regarding containment measures changed to focus on their impact on daily life. Policymakers should pay close attention to these changes in the public response to track the most vital needs of the population during the different stages of the pandemic and to address public concerns through timely and effective communication [45,46]. This can improve public compliance and engagement with containment measures and facilitate their implementation [47,48].

During COVID-19 resurgence in Beijing, a more targeted response strategy was applied for the high-risk population, and the general population was less impacted. Frequently discussed topics on Weibo during resurgence suggested that the lives of the public were returning to normal; there were no longer topics regarding discrimination and the shortage of supplies, and the overall negative sentiment toward all containment measures was lower during resurgence than during the initial outbreak. The shifts in discussed topics and the public sentiment might also be related to the reduced stringency of these containment measures. Concurrent with previous reports, stricter measures were followed by a more marked negative sentiment [16]. The public may become fatigued to containment measures when the pandemic resurges. In our study, public responses were more sensitive to the suspension of mass gathering events during resurgence than during the initial outbreak. Therefore, governments should balance the benefits of containment strategies to their impacts on daily life and the economy to formulate tailored response strategies during COVID-19 resurgence.

As more information on COVID-19 and its control has become available, governments have found alternatives to tailor their response strategies to its resurgence. Using the test-trace-isolate measure for high-risk populations, Beijing flattened the contagion curve and gained control over the pandemic within 1 month. Our study indicates that this targeted response strategy better matches public concerns. With the normalization of the pandemic, people cared more about their life and the economy. during the pandemic, such as the return to school or work, going to concerts, traveling, immigration, and sports events. The targeted response strategy focuses on high-risk populations and has less restrictions on most people's lives, which might help reduce the negative public sentiment during resurgence. A modeling study predicted that, compared with the community-wide lockdown strategy, the targeted response strategies would be less costly [49] and could help revive industries and the economy. China's economy data revealed that its gross domestic product decreased by 6.8% in the first quarter of 2020 during the complete lockdown but increased by 3.2% and 4.9% in the second and third quarters of 2020, respectively, with the test-trace-isolate measure targeting high-risk populations [50]. Therefore, more targeted response strategies should be promoted during subsequent COVID-19 resurgence.

Overall, the public reaction to the initial outbreak indicated a drastic escalation in public engagement, as evident from the increased number of negative sentiments after implementation of the lockdown, and, in comparison, targeted containment measures during resurgence gained more rational responses and greater support. The public expressed a high level of panic (21%) during the initial outbreak but virtually no panic (1%) during resurgence. Only 4% of the public expressed reluctance to be tested or quarantined during both the initial outbreak and resurgence, which is an important consideration for public health efforts to achieve universal compliance. The targeted test-trace-isolate measure received the greatest support among all 3 containment measures during the initial outbreak, and its support rate approached approximately 90% during resurgence. The evolution of public responses toward containment measures indicated that targeted test-trace-isolate strategies were more acceptable to the public. Governments should take public responses on social media into consideration, and develop more targeted test-trace-isolate strategies to prepare for the future resurgence of COVID-19.

### Limitations

This study has several limitations. First, Weibo is more widely used by younger people, and some users simply read Weibo posts but do not post their own content. Therefore, caution should be exercised when interpreting our findings in the context of the general public. We used Python to curate all relevant Weibo posts for analysis; therefore, our data are valid enough to reflect the opinions and responses of the Weibo users. Second, as an unsupervised text classification algorithm based on the “bag-of-words model” [38], LDA may lead to misclassification of Weibo posts. However, such misclassification is random; that is, it does not specify a certain direction (positive or negative) [51]. Therefore, it does not cause an interpretation bias in a comparative study. To control for this potential bias, we randomly sampled and manually classified Weibo posts to rectify for misclassification and improve accuracy. Future studies should perform real-time social media surveillance with more advanced machine learning techniques (eg, bidirectional encoder representations from transformers), and conduct long-term, multilingual, and multiplatform public response surveillance for the COVID-19 pandemic.

### Conclusions

Compared to the initial outbreak, the public became more rational during COVID-19 resurgence. The public expressed less engagement and less negative sentiment on containment measures, were more supportive towards containment measures, and shifted their focus to the impact of containment measures on their daily life or work during resurgence. Targeted test-trace-isolate strategies were more acceptable to the public. This study indicates that pandemic control strategies should be more targeted during subsequent COVID-19 resurgence, such as test-trace-isolate strategies targeting high-risk populations, to balance pandemic control and its impact on daily life and the economy.

## Appendix

Multimedia Appendix 1 Flowchart of data collection and preprocessing.

Multimedia Appendix 2 LDA outputs for each containment measure.

## Abbreviations

LDA: latent Dirichlet allocation
