# Supplementary figures and images for: Comparison of Public Responses to Containment Measures During the Initial Outbreak and Resurgence of COVID-19 in China: Infodemiology Study
Source: J Med Internet Res. 2021 Apr 5;23(4):e26518. doi: 10.2196/26518 (PMC8023317; doi:10.2196/26518)

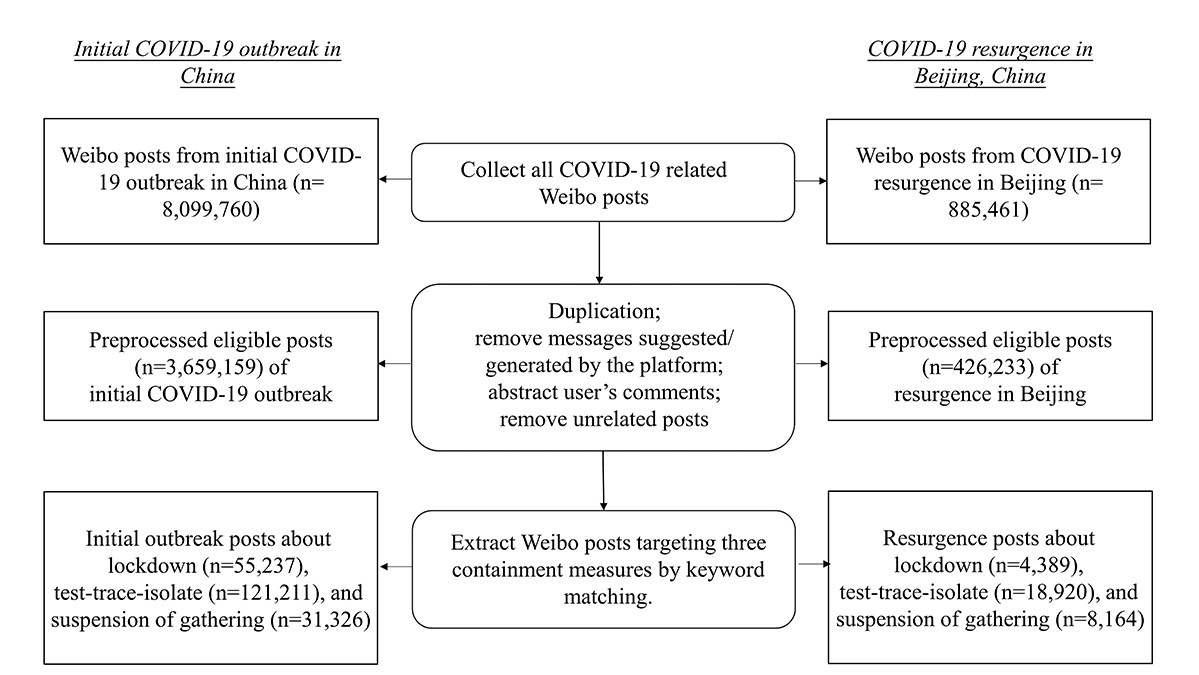

Supplement: Multimedia Appendix 1 [file jmir_v23i4e26518_app1.png]
