# Supplementary material for: Comparison of Public Responses to Containment Measures During the Initial Outbreak and Resurgence of COVID-19 in China: Infodemiology Study
Source: J Med Internet Res. 2021 Apr 5;23(4):e26518. doi: 10.2196/26518 (PMC8023317; doi:10.2196/26518)
Supplement: Multimedia Appendix 2 [file jmir_v23i4e26518_app2.docx]

Multimedia Appendix 2: LDA outputs for each containment measure

**CONTENTS**

[LDA output of lockdown measure during initial outbreak 2](#_Toc66308178)

LDA output of [test-trace-isolate measure during initial outbreak 7](#_Toc66308179)

LDA output of [suspension of gathering measure during initial outbreak 12](#_Toc66308180)

LDA output of [lockdown measure during resurgence 17](#_Toc66308181)

LDA output of [test-trace-isolate measure during resurgence 22](#_Toc66308182)

LDA output of [suspension of gathering measure during resurgence 27](#_Toc66308183)

### Initial outbreak – lockdown

Topic #0:

陕西 拜托 江苏 西藏 武汉 封城 三十 台湾 青海 南京 新疆 内蒙古 自由 症状 高原 自治区 徐州 不及 内蒙 苏州 没有 监控 澳大利亚 供给 医疗 十分 环境 恶劣 内地 部署 吉林省 正常 依旧 甘肃 秒拍 村镇 体育 西北 家家户户 说明 飞来 比赛 扬州 三十万 藏族 困境 不良 常州 双语 汉藏

Topic #1:

救援 世卫 目的 概率 检疫 指数 检验 热线 正月初一 预告 for 快马加鞭 出租 开局 此事 全线 大幅 营运 阳台 nba 公安局 陆陆续续 橙子 共享 单独 米面 废物 极限 遇难 先来 转自 百年 轨道 总部 武警 球星 进度 施策 截止 江门 租房 静下 车主 惊喜 印度 牵动人心 新陈代谢 人影 猖狂 讲述

Topic #2:

真心 帮助 谢谢 强制 执行 一方有难 伊朗 八方支援 早日 传来 必胜 强烈 南阳 医疗队 劝说 真相 网友 粉丝 公益 硬核 主任 名字 小镇 医生 分钟 医者 让出 餐饮 佩服 还给 史册 恶意 麻丘 风雨 关注 骂人 精力 掩盖 邯郸 载入 保卫 八方 人员们 第2 保安 华人 尊敬 疑问 返工 悲壮

Topic #3:

山东 云南 延长 一家人 图片 复杂 制度 火神山 父母 体现 兄弟 保重 昆明 查看 镇上 自救 涌入 西安市 效率 指挥 花园 初七 经营 至此 万家宴 具体 房东 东莞 说服 唯一 日期 澄迈县 亲情 赤壁 流泪 初八 京东 哄抢 全文 批发 要紧 姑姑 深夜 癌症 社会主义 大战 产品 漫延 财力 繁花

Topic #4:

影响 必须 经济 总动员 复工 操作 节后 错过 股市 预计 获得 资金 运行 拍摄 黄金 被动 历史 大事 愈发 is 事物 师傅 一千 节前 大幅 产业 早餐 决策者 精神 数学 科技 心灵 媳妇 雪上加霜 投资 位置 正轨 兴趣 畜禽 展示 天上 唯愿 农民 下跌 板块 保定 损害 小米 何地 开盘

Topic #5:

点亮 救助 一大早 一己之力 奉劝 视而不见 负起 冷血 没事儿 周口 拐点 人民政府 禁足 死活 年轻 纪念 五百万 反馈 环卫 再见 发给 盼望 气息 注重 麻麻 颜色 超越 主持人 考研 苦口婆心 泄露 拖到 一代人 真情 危险性 责备 率先 大海 普普通通 欢声笑语 捍卫 惴惴不安 高估 创伤 人头 飞速 艺术 宴席 whzf 利川

Topic #6:

高速 路口 公路 管制 告急 市委 国道 身影 大桥 入口 志愿 辖区 各县 收费站 一阵 货车 本溪 下雪 摸排 大道 切身 镇里 马国强 婚宴 冠肺 蕲春 一动不动 青山 烧烤 上鲁村 阿卡林省 宜昌市 汝州市 封心 睡衣 空降 奔跑 车牌号 徐峥 警车 上饶 十堰市 政局 新建 西湖 半路 灯火 路路 东风 查实

Topic #7:

上海 戒备 响应 超乎 号召 取消 旅游 公司 国家 疫情 酒店 退票 停运 江西 西安 广州 聚会 春节 航班 企业 通知 飞机 计划 期间 要求 机场 上班 那天 机票 员工 高铁 旅客 出发 在家 火车 登记 联系 今年 列车 免费 乘客 电话 转发 行程 铁路 结婚 四川 工作 出行 游客

Topic #8:

居然 记住 湛江 岳阳 自拍 高兴 无知无畏 折腾 伸出 灾害 夏天 现场 限度 第十 小岛 热度 死人 心思 牌照 封岛 湖南省 秘密 尝试 具备 切记 时时刻刻 四万 援手 帽子 渭南 就此 迷惑 喜悦 走街串巷 震撼 亲眼 施工 窒息 一对 男孩 好笑 七万 蛋糕 袭来 大同 召回 帮帮忙 我俩 班子 消防

Topic #9:

黄连 陕西省 独处 苏莱曼尼 预言 煽动 大有 疫战 当阳 封路封村 细品 酒吧 心境 一家子 坎坷 诗词 父老乡亲 本月 省亲 辐射 倒垃圾 曼巴 过世 遇封村 修建 下周 疼痛 小朋友们 机率 益伴 火箭弹 透透气 医务者 鹿晗 原文 使馆 bbc 天台 过敏 从业 全国一盘棋 l拾点八卦 连线 o双 难以置信 乌克兰 停车场 武漢人 嫌疑 立春

Topic #10:

响应 一级 启动 湖北 浙江 宣传 河南 事件 公共 卫生 突发 重庆 孝感 安徽 湖南 重大 重视 湖北省 黄冈 襄阳 城市 正好 zf 应急 周边 排查 确诊 病例 辽宁 已经 地级市 措施 省市 鄂州 宜昌 机制 县城 封省 口罩 作业 到位 停运 串门 发布 旁边 数据 救救 全省 人口 荆州

Topic #11:

民族 村村 急急 you 革职 线下 房贷 倒计 相隔 该当何罪 铜川 冬日 ll no fight life something if 玉米 有功 入户 fear awful short 正月初四 正月初三 nothing 货架 正月初五 粑粑 大帅 out 大豆 颁发 学员 证书 力差 穿越 农业部 谢天下 漫展 互动 整夜 麦当劳 佛教 杨凌 农科城 慈悲 钓鱼 分化

Topic #12:

动员 美国 信阳 以来 中医 生物 打击 战争 先生 奋起 拼杀 危机 生化 破坏 牵挂 全体 中药 散布 非典 抗击 解放军 武汉 禽流感 系统 怀疑 优势 自保 转基因 因素 武器 小路 派出 史无前例 悲伤 味道 农业 适应 涵盖 一刀切 要不得 今日 延误 恐慌 起来 发动 环境 压制 出手 丧失 断路

Topic #13:

病毒 武汉 疫情 肺炎 新型 封城 冠状 政府 感染 医院 负责 人员 确诊 没有 措施 隔离 传播 已经 全国 病例 目前 情况 医疗 患者 出现 时间 控制 物资 可能 防控 现在 问题 需要 应该 扩散 进行 传染 交通 各地 治疗 采取 信息 发现 病人 国家 有效 城市 人数 封闭 防护

Topic #14:

武汉 加油 病毒 疫情 新型 中国 肺炎 冠状 封城 人员 春节 人民 全国 医护 国家 一线 相信 希望 大家 社会 一起 平安 新年 抗击 致敬 健康 一定 微博 所有 工作 恐慌 生命 战胜 医务 人人 众志成城 新冠 这是 保护 人们 第一 人类 城市 感谢 今年 祖国 非典 快乐 生活 政府

Topic #15:

武汉 封城 现在 真的 没有 希望 不是 肺炎 看到 知道 大家 担心 已经 武汉人 觉得 很多 不要 出来 以后 消息 城市 事情 o武汉 严重 微博 今天 人民 病毒 起来 这次 过年 感染 地方 加油 新闻 可能 政府 应该 情况 害怕 全国 回家 野味 疫情 朋友 隔离 这种 之前 重视 一点

Topic #16:

出门 口罩 不要 病毒 新型 冠状 大家 尽量 洗手 接触 注意 一定 避免 场所 封闭 外出 保护 防护 地方 健康 家里 进入 预防 空气 减少 杭州 安全 做好 密集 卫生 佩戴 保持 在家 咳嗽 身体 感染 酒精 聚集 通风 相信 公共 提醒 症状 春节 人群 习惯 使用 传播 相互 第7

Topic #17:

视频 关闭 贺岁 公交 地铁 暂时 暂停 火车站 机场 通道 离汉 全市 客运 运营 市民 城市 轮渡 长途 不必 能否 离开 特殊 交通 壮士 原因 书记 通告 客车 娱乐 断腕 移动 另行 不要 武汉市 恢复 冠肺炎 宣布 仅次于 市内 厦门 长辈们 内外 为人 流向 每人 门店 剪辑 驻马店 报名 危害性

Topic #18:

科比 注定 爆发 世界 澳洲 伤害 原来 道德 老老实实 自然 人性 价格 几十 快快 灭绝 灾难 大自然 往外 有病 地球 就算 山火 大火 网约车 遭受 坠毁 吐槽 房子 宅家 蝗灾 良心 直升机 报复 千万 发生 老鼠 放松 空无一人 哄抬 导致 少将 成本 刺杀 烟台 地图炮 严重 一代 抵御 开头 坠机

Topic #19:

封城 武汉 全部 北京 全国 一定 所有 微博 盲目 下架 放下 空港 速递 l辰星 疫情 严重 百城空巷 河北 珍惜 庚子 驰援 山西 点赞 严防 千里 发表 蝙蝠 动静 前所未有 电梯 死守 福州 石家庄 讽刺 传到 广东 隔离衣 否认 艰难 一国人 校考 丽江 待命 三年 表演 家门口 宿州 无常 被迫 批判

Topic #20:

家人 电影 取消 朋友 想象 照顾 庙会 自信 严重 疫情 综艺 所有 推迟 天津 辟谣 家庭 后续 办理 开工 错误 一定 吉林 究竟 莫名 一两 病房 婚姻 模式 咨询 先后 领导班子 客人 带走 日子 给力 迟迟 封住 事后 特意 长春 助力 店铺 美帝 新冠 抓紧 例外 党员 活命 寻求 南宁

Topic #21:

武漢 台灣 野火 物資 通用 太太 捐贈 冠狀 苏贞昌 沒有 行政院长 足以 保護 因為 個n 罩給 請求 抗菌 宗教 貞昌 行政院 長蘇 政權 泯滅 怎樣 救武 醫療 漢嗎 確診 供應 救援 安利优 昌卻 蘇貞 殘酷 外銷 台灣人 极点 賣給 幫忙 綽綽 該盡 讓廠商 自產 願意 險峻 免費 醫疫症 期間 大年初八

Topic #22:

封村 可控 通报 啊啊 一边 行业 已经 肺炎 不止 封锁 建议 营业 大年初二 传染病 决定 代价 村民 唯一 求求 至少 国家 人士 没用 运动 汉口 进展 免疫 处理 目前 中心 不可 区域 抢购 测量 导致 提示 实验室 敬告 车辆 可能 肺部 可防 源头 发病 甲类 预测 漏网之鱼 病毒性 禁行 终究

Topic #23:

请求 自觉 樱花 天气 拜年 广播 封城 往年 乡村 愿望 武汉 千千万万 看看 各界 正月 希望 不再 第6 干部 福建 加重 春暖花开 美丽 婚礼 一会 各村 四月 家乡 烂漫 难得 闺蜜 无知 炫耀 生病 往常 期望 腊月 武大 治好 大都市 党中央 窗外 盛开 美食 高于 心痛 补给 好慌 山东省 实力

Topic #24:

完整 急诊科 笑笑 租客 云南省 don to 春风 监狱 面试 蛋壳 神经病 coronavirus 鼓舞 绍兴 刑法 阿弥陀佛 林晨 up实拍 说动 前10 按兵不动 创作 包机 好客 纹丝不动 up主 彝族 至使 一等 居心叵测 教练 跳舞 走漏 变好 岿然不动 高新区 楚雄 失信 王俊凯 new 长长 virus 搞来搞去 获得性 舞蹈 救国 哔哩 争气 顾忌

Topic #25:

市场 动物 海鲜 野生 华南 病毒 凝聚 法律 彻底 华夏 贩卖 濒临 交易 忙碌 买卖 来源于 销售 追究 造谣 录制 违法 非法 售卖 二月 存在 以前 果子狸 想尽 村里人 分配 奈何 学院 欢庆 入内 尸体 核实 采购 二十八 第十一 红十字 十万 杀害 执法 以户 绝灭 效应 规律 挖掘机 形象 噩耗

Topic #26:

迪斯尼 小区 开学 气死 延期 出口 公告 出自 lazzia 出租车 物业 集会 窗户 同日 报警 业主 举行 演出 只求 饿死 夜晚 举办 男子 萝卜 微博 大年初四 传奇 通知 博物馆 武汉站 挣扎 万达 风暴 沙发 专人 门票 国歌 演唱会 频道 艺人 发布 紧闭 中小学 行动力 即日 初十 县区 改期 土豆 商超

Topic #27:

口罩 没有 武汉 今天 家里 知道 一定 老人 封城 回来 希望 出门 每天 一下 过年 肺炎 在家 妈妈 关注 孩子 晚上 信息 独居 昨天 拜年 回家 基本 今年 超市 亲戚 上班 医院 村里 年轻人 早上 爸爸 人群 下午 明天 抵抗力 老家 感觉 爸妈 日子 购买 出去 发放 途径 来源 老人家

Topic #28:

建国 喊话 大陸 衡阳 月子 蜗居 特色 网点 手中 洛阳 沁园春 荆门市 羊肉 经典 他們 大年二十九 理白族 预报 應該 顿失 多娇 稍逊风骚 历史性 欠缺 耻辱 麻城 旋律 魔高 隔楼 万里 了却 开篇 试比糟 声名涛涛 窗吵 打车 疲惫不堪 道比 摆弄 雨过天晴 听罢 麻将室 交界处 炸鸡 获悉 心脏病 下放 方块 照单全收 张贴

Topic #29:

封路 非典 北京 海南 当年 这不 深圳 黑龙江 广西 封城 自行 意思 时期 日本 贵州 消失 郑州 空投 不准 痛苦 爱心 超过 鼠疫 病人 物流 不是 非常 严查 大地 泰国 武汉 扎堆 案例 相当 自私 车站 接近 乌泱 省际 往往 一万 十五 方便面 别人 属实 输入 病症 检查 体温 果断

### Initial outbreak – test-trace-isolate

Topic #0:

非典 隔离 本来 害怕 口罩 当时 现在 每天 北京 面前 回家 学校 新闻 回来 喷点 上班 出门 肺炎 过年 这次 体温 没事 感冒 感觉 发烧 那时候 起来 回去 家里 知道 同学 发现 放假 后来 海南 就要 严重 告诉 当年 不用 有点 外地 记得 电视 担心 不是 正常 经历 亲戚 没有

Topic #1:

这种 局部 原体 抗病 网友 救治 讨论 思考 全力 生气 买单 黑暗 骄傲 志愿者 奉献 浪费 自愿 究竟 微信群 敌人 争取 武装 担当 全副 安危 命令 抗击 无关 医者 防线 业主 死神 缺少 真假 该死 相互 辱骂 从事 生死 信仰 报酬 丝毫 可靠 局势 手里 画面 出征 隔离点 艾灸 走向

Topic #2:

口罩 不要 出门 大家 隔离 做好 防护 自我 一定 洗手 保护 注意 尽量 消毒 预防 物资 在家 家人 工作 拜年 地方 外出 医用 负责 家里 人员 安全 聚会 身体 自行 防范 健康 主动 减少 朋友 公共 重要 人群 疫情 恐慌 酒精 提醒 最好 通风 免疫力 提高 n95 措施 密集 避免

Topic #3:

病毒 冠状 新型 控制 希望 成功 热搜 明天 感染 目前 病例 新闻 发现 新增 武汉市 检测 呼吸道 接触者 肺炎 定点 死亡 医学 入院 中心 累计 核酸 治愈 正在 报告 密切 出院 疾控 黄冈市 收治 稳定 孝感市 接受 截至 检验 机构 三口 专家组 卫健委 阴性 阳性 确认 温州 北京 追踪 危重症

Topic #4:

武汉 没有 隔离 现在 真的 武汉人 知道 回来 不是 政府 已经 排查 封城 确诊 城市 出来 重视 肺炎 很多 一下 人员 不要 宣传 应该 地方 情况 严重 病例 大家 中国人 潜伏期 自我 觉得 起来 口罩 接触 不说 问题 全国 感染 看到 传染 出去 根本 信息 这种 之前 离开 回家 求求

Topic #5:

疫情 病毒 控制 肺炎 武汉 传播 新型 全国 可能 国家 冠状 防控 感染 应该 没有 这次 现在 措施 政府 有效 已经 时间 新冠 需要 隔离 出现 目前 不是 情况 扩散 人员 非典 人口 及时 造成 相信 恐慌 sars 传染 感染者 爆发 病例 发现 中国 问题 信息 医疗 存在 春节 一定

Topic #6:

传染病 疫苗 卫生 人类 预防 自然 措施 研发 公共 拥有 管理 疾病 采取 时间 事态 利用 控制 鼠疫 良好 针对 系统 列为 大规模 甲类 获得 再次 献给 肺炎 持续 sars 监测 我国 设施 流行 临床 提供 上涨 应当 交流 完善 科研 建立 分离 世界 机制 进行 前提 拐点 长期 天下

Topic #7:

孩子 返乡 河南 排查 父亲 一级 打开 母亲 形势 超级 严峻 启动 坐标 惊慌 父母 主动 退票 起床 起码 台湾 村民 今早 封村 西藏 休假 警告 耽误 妻子 腊月 焦虑 南阳 十五 刻不容缓 丈夫 工资 指引 宝贝 黑龙江 报备 肾病 做起 开会 攀升 调配 华中 车票 人中 传染者 珠海 赛跑

Topic #8:

领导 拜托 单位 广州 春晚 除夕夜 今天 到达 春暖花开 宜昌市 干部 火神山 无辜 神农架 签到 雷神山 天灾 脑瘫儿 博主 宜昌 滞留 可想而知 黄石 元宵节 留守 力所能及 快手 人祸 央视 份子 设计 天门 去向 晚会 北海 安徽省 牵连 帽子 三亚 募捐 封岛 首都 急救 党委 荆门 产业 水深火热 拍摄 党中央 青年

Topic #9:

接触 病毒 动物 隔离 传染 野生 症状 冠状 密切 观察 新型 病例 居家 感染 野味 卫生 避免 治疗 别人 情况 可能 肺炎 健康 有点 类似 建议 传播 家庭 必要 具体 需要 个人 途径 咳嗽 大家 武汉 打喷嚏 疾病 已经 人类 国家 sars 组织 洗手 控制 安全 市长 保持 特别 防控

Topic #10:

社区 在家 春节 微信 假期 强烈 街道 西安 生命 新加坡 建议 据说 随意 警察 出来 接诊 上门 直面 要求 仙桃市 进去 手术 垃圾 承受 随便 无法 乡村 排队 关门 安排 莫瞎串 老爸 安静 杀死 门口 昆明 卫生部 泄露 诊所 第一 到处 意到 电视剧 帮帮 非洲 服务 脱离 大爷 鄂州 第四

Topic #11:

市场 海鲜 华南 混淆 时间 疫区 交通 香港 工具 小时 关闭 统计 人士 乘坐 医学 自我 改变 明显 好转 分钟 失去 记录 易感 同济 选择 全部 建设 温度 直接 以上 英国 最终 关键 留观 活动 费用 预计 运动 能否 高温 左右 等待 恢复 加重 急诊科 不足 方向 环境 超过 这才

Topic #12:

没有 知道 真的 现在 不是 觉得 很多 今天 家里 每天 妈妈 事情 在家 可能 朋友 看到 家人 生活 生命 别人 感觉 其实 最后 亲戚 时间 大家 有人 只能 一点 爸爸 担心 身边 东西 特别 第一 过去 恐慌 自我 严重 最近 突然 一下 医生 心里 恐惧 应该 理解 非常 人们 好好

Topic #13:

希望 控制 武汉 加油 疫情 人员 得到 医护 看到 肺炎 中国 平安 大家 病毒 所有 早日 一线 相信 人民 新年 一定 不要 全国 尽快 健康 消息 新闻 起来 感染 真的 微博 能够 新型 病情 已经 保护 国家 一起 过年 平平安安 前线 野味 每天 有效 早点 家人 第一 春节 致敬 快乐

Topic #14:

开学 去世 科比 大学 延迟 外婆 陕西 重新 国民 村庄 时艰 学校 共克 派出所 赢得 舅舅 外公 日期 心慌 澳门 菜市场 只求 改造 收留 表哥 管用 巨星 中西医 隐形 聊聊 共享 勇士 原始 坠机 实锤 赣州 申报 坐地 安危事 师生 执行力 没钱 青山区 保住 原有 召回 中断 舅妈 连花 下旬

Topic #15:

症状 治疗 病毒 出现 感染 发热 患者 抑制 咳嗽 呼吸 肺炎 隔离 毒药 感冒 黄连 表现 目前 临床 诊断 没有 注意 中医 药物 预防 免疫 轻症 发现 人体 呼吸道 病情 体温 山东 困难 乏力 发烧 自我 使用 系统 方法 主要 指定 发病 进行 下降 就诊 病例 需要 干咳 双黄连 危害

Topic #16:

湖北 市民 萧山 免费 防范 强制 证明 意识 外逃 提示 害人 进主 白衣天使们 喜乐 自救 有事 害己 聚众 人流 农民 死角 对方 up主 温馨 一部分 节目 盲目 允许 走访 可恨 觉悟 药房 咸宁 合肥 发起 老人家 一同 敬意 薄弱 伙伴 政府 恳求 吴倩 互联网 东莞 白细胞 武汉籍 保守 英雄们 带上

Topic #17:

隔离 武汉 回来 在家 自觉 小区 举报 学生 距离 湖南 串门 重灾区 直接 男子 山西 澳大利亚 测试 表示 老婆 进行 一起 大面积 女子 勇敢 大儿子 作业 收入 等待 书院 正在 肺炎 父母 开启 女士 期间 十八 彭城 扬州 不准 子女 公里 某某 女足 您们 老公 探亲 情分 飞往 消毒剂 千里

Topic #18:

联系 酒店 转发 提供 捐款 回复 点赞 服务 平台 行程 保障 暂停 完成 地图 取得 价格 坚持 机票 商家 计划 沟通 入住 预定 结婚 大数据 政策 协助 几千 支付 升级 体现 宣讲 链接 遗憾 遵守 客人 身份证 资料 荆州 淘宝 岳阳 获取 块钱 官方 吐槽 举国 感谢 衷心 购物 亲情

Topic #19:

钟南山 院士 目前 特效 常见病 药物 研究 传人 毒性 有用 教授 没有 非常 随州市 人情 采访 南山 村干部 直播 差不多 tm 飞沫 后期 共度 隔离 高龄 错过 呵呵 安全感 阻挡 普通 繁花 阜阳 呼吸机 拥抱 常常 准备 罪人 无处 安抚 开建 行走 白岩松 自律 恐慌 责任心 活埋 多日 争分夺秒 坐镇

Topic #20:

今天 已经 昨天 晚上 一家 公司 电话 早上 下午 回家 发烧 上班 小区 大概 体温 家里 打电话 咳嗽 醒来 凌晨 同事 口服液 通知 我家 吃饭 明天 睡觉 接到 上午 本人 要求 昨晚 准备 天后 手机 舒服 奔赴 决定 感冒 继续 排队 终于 中午 某一天 一起 有点 大连 消息 邻居 半夜

Topic #21:

医院 隔离 肺炎 患者 确诊 医生 武汉 病人 疑似 治疗 感染 医疗 病房 人员 发热 观察 检查 自行 上海 医护 护士 接受 门诊 床位 人民 住院 病情 资源 普通 重症 发烧 杭州 情况 防护服 家属 小时 出院 无法 工作 医务 居家 回家 一线 只能 支援 求助 及时 病毒性 企业 交叉

Topic #22:

美国 多么 操作 法国 当下 阐明 公告 关注 国立 倡议 亲朋好友 恶心 系列 他妈 在于 官员 研究所 心痛 撤侨 可怜 反思 任务 善待 极其 热门 动向 民间 华人 安阳 岁月 智慧 今日 亲爱 景点 祷告 阻隔 婚礼 一辈子 服务者 世纪 未来 祈求 打倒 静好 所长 感染症 日本人 空军 贸易 来自

Topic #23:

取消 区别 祖国 喷剂 安徽 江苏 计划 年夜饭 头条 三十 全部 产品 十四 抓紧 够用 潜江 up 这下 出行 景区 初七 公公 抖音 河北省 港口 妈的 逃走 用户 省内 青海 苗族 劝返 驾车 交警 纪念 丁香 巴黎 封省 孩子们 运营 镇长 点开 贺岁 交谈 恩施 白菜 后援 宗教 新浪 芬芳

Topic #24:

这次 没有 骗子 真是 隔离 故意 到处 女儿 爷爷 徐州 国人 新春 援助 成本 初一 湖北人 奶奶 隔离期 良心 入户 车厢 目的 文化 及早 热闹 庚子 简直 佳节 小儿子 东北 好不 国难 往往 位置 伸出 淡薄 协调 坚定 文明 与共 成长 热线 外国人 上海人 冠肺 援手 漫长 将近 信念 终将

Topic #25:

流感 谢谢 麻烦 中药 细菌 大自然 荆门市 消除 公卫 过后 背后 治好 分享 跟着 医疗队 明白 河南省 涉及 爱护 重要性 醒醒 没用 为此 南昌 体内 来讲 抗生素 做错 间接 欧洲 说真的 内容 菩萨 炎症 嘱咐 每次 一下 众生 发病率 主要 回向 高血压 能量 领导班子 功德 西班牙 大理 小说 松懈 控制

Topic #26:

中国 事件 紧急 国际 卫生 国家 公共 责任 突发 宣布 家中 要求 处理 强制 部门 执行 请求 设备 湖北省 调查 荆州市 行为 全球 规定 集体 申请 物价 l中报 联合 工人 十堰市 上级 恩施州 林区 交易 发布 开工 咸宁市 透露 加拿大 法律 秩序 生产 权利 股市 失职 重大 集团 反映 土地

Topic #27:

歧视 恢复 白衣 天使 延长 员工 生活 影响 输入 信阳 大约 勾结 官商 长期以来 但愿 行业 新冠肺炎 全体 自由 平凡 小朋友 物业 驰援 地球 实现 沉重 受到 抢救 职业 算了 低于 危机 进入 义务 营业 状态 剩下 德国 对比 享受 会议 客户 内地 杂志 回归 o首 比例 意大利 伤害 不能不

Topic #28:

视频 微博 登记 快点 肺炎 旅客 车辆 机场 力度 工作 武汉 进行 秒拍 加大 体温 团队 揪心 加强 公安 进出 短缺 世界 疫情 管控 离汉 潜江市 同行 全面 来自 嫌弃 至今 负面 在线 改签 传递 火车站 烟花 赞颂 赞叹 为人忌惮 弹幕网 l哔 比赛 测温仪 活禽 辖区 配备 设置 坚决 日记

Topic #29:

黄石市 冠肺炎 学学 武漢 道路 五行 护栏 桥梁 以此 涉嫌 实录 至此 立春 含氯 冠狀 唐山 厂家 不锈钢 麻将馆 安息 栏杆 工程 急急 乙醇 食欲 首席 脖子 撤档 横幅 不妨 分餐 球员 海鲜店 真情 标语 活体 煮沸 救护 菜场 土耳其 疑染 吸收 氯仿 灯光 乙醚 生产 推动 夜间 师傅 衢州

### Initial outbreak – suspension of gathering

Topic #0:

推迟 开学 时间 上班 学校 延期 武汉 人员 肺炎 流动 放假 应该 大学 学生 希望 减少 全国 延迟 学习 国家 不是 情况 建议 感染 通知 上学 状态 觉得 高校 现在 高考 等到 看看 乐观 防控 正常 初七 大学生 春运 整体 初六 老师 寒假 一下 就要 强烈 可能 考虑 家长 暑假

Topic #1:

延期 举行 演唱会 比赛 疫情 南京 进行 通知 体育 河南 公告 cba 待定 联赛 时间 云南 球迷 日常 一部分 亚洲 女足 定于 延后 可能 中超 羽联 昆明 成长 联合会 驰援 俱乐部 直播 教育局 队伍 嘉宾 训练 足球 赛季 亚冠 补课 兄弟 球员 福气 慎重 表示 黑龙江 携手 总局 足协 赛程

Topic #2:

饭店 呼吸 中医 冬天 发抖 火神山 瑟瑟 排除 年初一 滑雪 甜姐 拜年 ktv 离世 智慧 小汤山 婚期 五行 百度 饭局 亲情 逃脱 看书 阜阳 减肥 搜索 温泉 袭击 干预 不容 暗示 刷出 官商 c位 腹泻 零时 自如 靠边 祝余 华南余 wcnm渣 招聘 警报 到时 忧虑 死去 疫时 开集会 反正 抗新型

Topic #3:

酒店 武汉 春节 珠海 取消 携程 肺炎 申请 提供 来自 只能 原本 证明 家人 组织 发热 一共 麻烦 硬着头皮 做好 知悉 防控 外省 措施 增添 退款 订单 客服 免费 机票 预订 全额 民宿 飞猪 房东 消费者 平台 预定 协会 vivips 用户 入住 退订 投诉 住宿 美团 退改 大酒店 星城 长隆

Topic #4:

国际 演出 世卫 会议 pheic 英国 意大利 who 街头 各国 年会 中心 停飞 爱心 国籍 辖区 大阪 灯会 与共 构成 繁花 主办方 大年初五 当局 中山 安徽省 视为 结局 东湖 安阳 考量 罗马 场馆 伊朗 货物 脱欧 所需 暂停键 中止 癌症 权力 年初六 早报 倡议书 文艺 土耳其 停航 ho1340 身亡 教堂

Topic #5:

口罩 出门 取消 尽量 活动 超市 今天 明天 地方 快点 打算 消息 酒席 街上 出去 不用 文章 记得 家里蹲 回国 本来 事儿 集会 实施 看到 上班 外卖 羽毛球 不当 加拿大 获取 中老年人 宗教 一点点 开业 采购 断货 顺便 到位 少女 春晚 朋友圈 口服液 淡薄 京东 没关系 正月十六 双黄连 姥爷 口岸

Topic #6:

资格赛 拳击 高山 雪联 大河 体育馆 翠波鸟 巢穴 电子 祷告 此举 奥运 替换 剧增 求主 延庆站 b组 大洋洲 耶稣 工作组 领衔 国家队 丰田 洪山 笼子 发言 基督 房价 开幕 间隔 筑巢 山西省 奥委会 塑料袋 冬奥会 怜悯 测试赛 华仔 赞美 剧方 延时 划入 闭店 延庆 亚太 首场 预选赛 王子 巴厘岛 大河南

Topic #7:

科比 接触性 harper 胃肠道 眼结膜 称为 粉丝们 大兴 咖啡 直升机 遇难 属性 佛罗伦萨 坠毁 热气 横幅 使馆 选手 伊拉克 大使馆 开诊 火箭弹 获悉 哭泣 商丘 筛选 决策者 后方 群防群治 旌坞村 口腔科 悼念 星宿 文体 榜样 暴跌 蝗灾 农户 八脉 奇经 运势 印度尼西亚 不懈努力 悬挂 刑拘 先驱 七点 阳历 失火 逝者

Topic #8:

限制 广大 客户 取消 保险 医疗 项目 住院 举措 针对 肺炎 线上 内蒙古 十四 治疗 药品 西藏 应急 启动 滴滴 理赔 陕西 重大 等待 定点 ncov 购买 费用 关怀 诊疗 发散 赔额 冠肺炎 人寿 报销 武昌 详情 大江 青海 免责 自治区 网约车 汉阳 原有 焚烧 通道 眼下 赔付 投保 绿色

Topic #9:

景区 文化 庚子 取得 冠狀 爱护 俄罗斯 干部 旅游局 雷神山 独处 满满 特斯拉 法国 同舟共济 滑雪场 正月十七 村委 model 消耗 资讯 雾霾 印度 活动 教师 中國 尝试 实践 公务员 正规 出入境 相反 告急 女子 管理局 景象 寒冷 智能 焦点 禁足 清理 男女 進行 云南省 武汉市 猪肉 签注 封闭 恭祝 牛肉

Topic #10:

微博 视频 朋友 sars 武汉 成为 疑似 阅读 体温 急救课 电视 广播 印象 春天 上课 湛江 初三 小学 作业 深刻 幼儿园 推送 那年 明年 测量 校园 娱乐 有没有 公众号 定金 公交车 小区 中药 中考 循环 年级 l宇宙 第四 沉浸 上去 教室 家长 参观 夏天 非典 记忆 初中 党员 真假 一日

Topic #11:

疫情 取消 照顾 病毒 贺岁 新型 肺炎 冠状 人员 空港 工作 大家 健康 武汉 安全 所有 退票 响应 决定 医院 期间 防控 活动 原定 计划 通知 时间 确诊 做好 国家 春节 公司 情况 旅游 出行 休假 减少 政府 航空 目前 相关 防护 行程 感染 是否 旅行 特殊 时期 避免 抗击

Topic #12:

黄连 博世 杀业 裁员 戒杀 圣号 炎症 球星 欧元 印光 感召 横事 贼寇 橘子洲 中西医 零部件 血液 过山车 几近 柒闲 疑因 l竹林 耳鼻喉科 梁武东 苦海 泛泛 享尽 九天 焰火 核算 永不 淮北 驻华 宿世 世沦 等灾 受完 生出 效验 倒行 乃是 见闻者 重恩 悉发菩 诚持 放生者 起死回生 同生 恶业 下济

Topic #13:

家人 全国 北京 所有 封城 负责 戒备 盲目 放下 想象 迪斯尼 综艺 疫情 一边 家庭 山东 转发 临床 钟南山 武汉 相互 内地 点赞 伤害 封路 转告 呼吸道 职业 显得 不要 一定 分析 协和 医学 破坏 床位 院士 买单 直至 股市 病房 人情 亲人 晚会 开端 食品 痊愈 停工 客车 事业

Topic #14:

进入 可能 高峰 天气 达到 机制 目前 肺炎 感染 武漢 之外 病菌 停留 小心 预测 短暂 上述 判断 无限期 播出 台北 疫情 季节 大力 纪念 尽早 长期 梦想 高峰期 发表 拐点 给出 成为 大概率 为止 航班 以上 台灣 桂林 流程 房贷 非洲 线路 汕头 现场 分别 免除 上旬 检验 贷款

Topic #15:

武汉 没有 希望 疫情 现在 肺炎 加油 大家 真的 取消 不要 自信 不是 很多 已经 看到 知道 这次 口罩 人员 春节 病毒 国家 严重 今年 相信 停止 家里 感染 拜年 一定 在家 聚会 隔离 全国 起来 可能 中国 重视 非典 传染 人民 医院 觉得 每天 第一 医生 过去 政府 封城

Topic #16:

举办 凌晨 赛事 频次 捐款 居家 球队 组委会 面临 上面 仅仅 上海市 徐州 村庄 元宵 小镇 春季 贸易 万事如意 穿着 世界杯 无锡 走上 早餐 召必 马拉松 时艰 丈夫 造成 警醒 致以 lpl 猜测 伦敦 共克 透露 媳妇 并未 神州 走亲 无非 灿烂 周到 退役 田径 第14 闺女 留在 门前 妻子

Topic #17:

病毒 新型 冠状 中国 影响 肺炎 暂停 航班 传播 美国 感染 日本 世界 公司 引起 停止 目前 出现 导致 签证 恢复 企业 治疗 生产 sars 扩散 研究 行业 正在 工作 病例 预防 原因 开工 韩国 部分 已经 控制 环境 空气 旅游 带来 造成 疫苗 工资 预计 收入 身体 数量 消息

Topic #18:

大家 取消 平安 健康 婚礼 今年 安全 过年 拍摄 行程 注意 第九 武汉 被迫 最近 新人 人流量 赶快 婚姻 暂时 民政局 出差 口罩 到处 制片 所有人 婚宴 在一起 恳请 小心驶得万年船 跑步 大年初四 爆竹 到岗 遗憾 人心惶惶 想念 脱离 唯一 新人们 烧香 地方政府 建成 正月初六 广元 the 世音 心思 来宾 能量

Topic #19:

产妇 辛有志 白菜 马某 周六 开店 贫穷 多娇 党中央 取名 沁园春 省亲 气节 顿失 揭阳 魔高 濮阳 断交 o假期 稍逊风骚 经典 试比糟 声名涛涛 道比 邪金 正月初五 产科 分娩 击败 施行 冠军 房地产 一亿五千万 厚重 子孙 赛场 预留 决赛 倾尽 生火 战毒 产后 母女 生命力 采样 团队游 易经 离卦 魔千 掏出

Topic #20:

暂停 武汉 停止 营业 公共 交通 动物 城市 疫情 场所 野生 措施 人类 地铁 关闭 公交 卫生 采取 湖北 运营 禁止 暂时 市场 组织 市民 全市 人口 宣布 客运 机场 经济 病例 肺炎 活动 湖北省 确诊 火车站 所有 停运 影视 各地 中国 封城 武汉市 开放 进行 大型 排查 通道 期间

Topic #21:

考试 校考 老师 教育 考生 学院 真的 高三 放过 艺考 打牌 复习 艺考生 大地 集训 重启 美术 考点 参加 理论 驾驶人 眼镜 院校 小说 苦口婆心 备考 支队 美院 联防 庚子年 理想 年头 同学们 期末 科目 画室 二月份 白费 化解 超越 复读 考完 要命 很难说 联控 睁开 军方 空无一人 送到 保山市

Topic #22:

假期 延长 春节 建议 复工 返程 肺炎 爆发 防控 今年 可能 时光 卫健委 能够 节后 大火 吓人 客流 返城 同意 鼠年 刘德华 迹象 悉尼 目标 出游 澄迈县 消退 板块 大面积 唐人街 绍兴 可怕 受到 反弹 苏州 不时 岁月 囧妈 战疫 管理层 探案 派遣 来去 天空 iphone 广场 正值 愈演愈烈 不宜

Topic #23:

孩子 老人 捐赠 机场 抗击 开工 公园 面对 英雄 冷清 红包 架次 不得 开门 三亚 o疫情 东北 来到 初九 援助 谢谢 为此 腾讯 高级 民航局 抵达 浦东 经停 起飞 监控 正能量 符合 主演 传统 复工 德国 青年 汉莎 盛世 这位 餐馆 官微 现有 硬核 复盘 抵制 阴影 男女老少 方向 家宅

Topic #24:

宣布 香港 另行 物资 天津 业务 抑制 供应 通知 武汉站 列车 交警 办理 行政 新华 商城 兰州 保留 面试 对称 巡回 平稳 活動 服務 明日 決定 喧嚣 募捐 影響 纳入 內地 收回 监狱 调度 储备 漫延 因应 下月 周三 停靠 澳門 车队 朝鲜 乘务员 com 聪明 联航 组建 防线 国考

Topic #25:

电影 全部 取消 严重 庙会 一定 下架 上海 武汉 疫情 超乎 关闭 速递 l辰星 百城空巷 谅解 敬请 撤档 演员 家待 博物馆 上映 影院 祝福 闭馆 故宫 对外开放 给到 电视剧 高分 囧妈 剪辑 工厂 景点 环节 特此 掉以轻心 博物院 徐峥 剧场 票房 剧院 夺冠 院线 抽奖 电影院 一波未平 一波又起 贺岁档 电影票

Topic #26:

精品 众生 失能 ck xba chaumet 享日 大师 九旬 黄浦区 ace2 帝国主义 目地 透析 品种 赠送 亲和力 念佛 区委 信访 生物学 罢工 生命科学 住房 慈悲 吃惊 回向 批次 制造者 巴斯德 合成 心机 走入 rbd 构域 英文版 之余 灭绝人性 郝沛 误区 皮细胞 围剿 相互作用 卓越 钟武 愤怒无比 功德 浪漫 商务 经济舱

Topic #27:

青岛 办公室 设备 萝卜 糖球会 海云庵 基金会 十六 救救 接种 村村 正月十四 漏网之鱼 贵州省 情愿 回城 歉意 华米 魅族 头痛 间谍 演唱 下周 好福康安 人情淡 via 有感 冻结 to it 正月初 潜伏者 网络格 分化 要害 大邱 财神 律师 江原fc 大邱fc 尚州 尚武 例数 抢光 劉德華 温度计 低温 一言难尽 門票 声称

Topic #28:

科比 青春 lisa 人民 影视剧 欢迎 节目组 首都 采访 坠机 心酸 限行 断章取义者 卫视 高发期 巨星 批评 有幸 唯独 滋味 搞笑 nba 由此 普遍 哈哈哈 国贸 金融 愉快 房租 传奇 收到 直飞 快手 大本营 正月初九 玩儿 沙发 筛查 绝大部分 战略 出逃 极少 网点 正当 周五 陨落 临汾 关切 姐夫 称作

Topic #29:

取消 今天 家里 过年 回来 肺炎 没有 妈妈 本来 在家 回家 晚上 亲戚 知道 准备 计划 上班 昨天 爸妈 明天 现在 已经 口罩 真的 每天 爸爸 登记 一起 终于 打电话 聚餐 下午 通知 电话 有点 早上 结婚 一下 全部 感觉 孩子 担心 起来 手机 好多 新年 日子 聚会 我家 好好

### Resurgence – lockdown

Topic #0:

北京 新发地 全国 地方 人民 菜篮子 供应 农产品 价格 防疫 可能 不是 信心 物资 本土 愿意 仅仅 面对 清楚 确保 成果 六月 工作 连夜 真的 肯定 新发 回到 来说 小学 炸锅 当年 正确 考验 认识 牺牲 这不 时间 相关 分流 水平 波动 京城 微信群 物价 估计 哄抬 概念 华北 执政

Topic #1:

回头 国内 以为 抢光 德国 底下 好像 法国 桌子 身上 进行 形势 开学 看见 取消 总是 封城 严峻 影响 居然 刚刚 正常 个人 患者 疫情 严密 观察 调查 采样 新增 北京 消毒 进一步 所在 监测 目前 活动 地坛 密切 管理 医学 追踪 应检 封闭式 做好 新冠 今日 医院 小区 场所

Topic #2:

封城 武汉 疫情 歧视 看到 武汉人 完全 期间 手绘 河北 山东 孙子 tm mv 客户 小时 天津 并没有 完成 一下 儿子 没错 帽子 大约 山西 healy 出新 音乐节 fran nme 捞钱 拉踩 主唱 立判 高下 落井下石 举报 不止 宁夏 攻击 河南 推到 地域 敏感 地图 哥哥 甘肃 他妈 开玩笑 肺炎

Topic #3:

北京 刚刚 响应 运营 正常 接单 家暴 困难 玉泉路 高速 在于 商店 关闭 注重 好事 高效 商业 未曾 他妈 路透社 独善其身 集训 音乐节 下次 动物 大年初三 新京报 对面 全国 消杀 空气 周到 全阴性 偶然 全额 测温 卖家 最低 展现 摸底 无数 确保 上街 慌张 组建 协调 助力 顺风车 董事长 海鲜市

Topic #4:

资源 电影 旅游 影响 出境 产品 疫情 消费 质量 电视剧 价值 小小 境外 每次 企业 一部分 好看 高速 围绕 电影院 之后 小时候 旅行 憧憬 尾号 逐渐 压力 转化 喜欢 欺负 软件 片段 西北 持续 人们 人生 服务 缓解 生计 第一 境内 父亲 热闹 国内 无法 好多 方向 家暴 漂泊 过度

Topic #5:

金融 到底 广告 受害人 高峰 绝望 程度 国家 一再 自私 退还 特别是 中央 受害者 资产 梳理 翻倍 打击 消耗 中国 平台 早已 没完 限制性 救治 上车 全额 落实 支付 祸害 叠加 深受 发文 实事 治理 处置 无处 伤害 无视 改革 多久 支援 放弃 几十 决心 下去 访客 形成 大量 达到

Topic #6:

北京 疫情 封闭 小区 检测 核酸 没有 现在 响应 今天 已经 新发地 隔离 大家 工作 不是 人员 知道 生活 希望 很多 政府 真的 管理 社区 快递 时间 昨天 这次 口罩 需要 感觉 每天 出来 问题 过去 通知 加油 医院 二级 风险 起来 看到 地区 正常 所有 确诊 可能 一下 回家

Topic #7:

平凡 珍惜 vlog 小姐 l王 来之不易 成都 看看 以前 周末 小事 家宅 下调 如果说 日子 旅行 寻找 跟随 当下 往常 级别 尝试 每个 响应 疫情 注意 可能 恢复 视频 微博 一起 时间 市场 央媒 应急 调整 人员 报告 肺炎 新冠 检测 阳性 环境 报道 病例 看到 封闭 出现 新增 从业

Topic #8:

疫情 响应 号召 王一博 大家 期间 视频 微博 聚集 扎堆 北京 粉丝 市场 本人 安全 呼吁 添麻烦 当然 口罩 保持 别人 国家 封闭 蔬菜 防控 保护 新发地 减少 配合 水果 洗手 需要 早日 不要 距离 防护 工作 注意 隔离 批发 做好 二级 加油 支持 企业 继续 影响 应急 希望 上涨

Topic #9:

肖战 购物 商场 复学 作品 应援 做为 晚饭 战战 体温 爱粉 遇见 大门 助力 爷爷 偶尔 原定 抹黑 这时候 声明 入口处 闲逛 摊主 城管 热点 安康 亏损 顾客 治安 摆摊 进货 为此 维护 安徽 认真 推进 小学 下来 活动 人员 心情 小区 进入 中心 封闭 今天 下午 管理 社区 意外

Topic #10:

病例 丰台区 工作 学校 进行 检测 美国 疫情 人员 孩子 单位 确诊 患者 之前 医院 原因 口罩 环境 新发地 暑假 隔离 核酸 发热 惊呆 这是 要求 正在 part 开展 悄悄 遗留 带走 肺炎 出门 眼前 排查 通知 昨天 接到 物品 北京市 儿子 接触史 暴乱 开学 第一 采集 教室 骚乱 家长们

Topic #11:

北京 人员 防控 市场 疫情 检测 工作 北京市 新发地 管理 进行 病例 核酸 社区 措施 封闭 确诊 响应 小区 肺炎 二级 相关 级别 周边 调整 应急 严格 要求 恢复 场所 批发 全部 暂停 新闻 公共 高风险 医院 新冠 目前 通知 街道 所有 排查 出现 管控 做好 全市 居家 落实 全面

Topic #12:

网络 电梯 订单 上门 快递员 淘宝 单元 周期 味道 着火 花园 商品 单价 恶劣 饿了么 配图 楼道 送货 时间 好像 打扫 前年 阳台 生食 关闭 转移 吃喝拉撒 鸡头 喂食 圈养 风吹雨晒 全栅栏 四周 偶然 接单 商店 运营 存在 电子版 胶皮 小事 彻查 暂时 结束 半月 天猫 预期 中旬 不足 滚滚

Topic #13:

出院 手术 离家 吃吃喝喝 顾客 家里人 二十几 格外 当日 一月 构成 看似 医院 平日 华南 朋友圈 幸运 战场 最近 海鲜 安静 全封闭式 派出所 进行 好受 新冠 战士 管理 病例 封城 年初 不知 没有 无数 市场 下来 疫情 肺炎 所在 小区 确诊 新增 封闭式 生命 北京 出发 选择 夏天 其实 患者

Topic #14:

平台 人民 自觉 舞蹈 疫情 自愿 艺术 练功 喜欢 白衣 医生 非常 时期 抗击 值得 全国 专业 天使 奔赴 命令 全民 力量 用户 痛苦 熟悉 民警 享受 动员 春节 上线 庚子 亿万 商家 经常 兴趣 出门 视频 青年 每个 热爱 迅速 肌肉 带领 疫区 文章 凝聚 湖北 聚会 战场 原因

Topic #15:

地铁 火车 其实 应该 主要 严重 人流 封闭 华南 空间 武汉 传播 新发地 疫情 之前 露天 想法 起来 集市 周转 汉口站 路线 班次 亏损 搭乘 起点 常识 处在 半封闭 屁用 盒马 到达 便利 顶替 缅甸 线路 资源 数量 交集 运营 容纳 质量 价值 消费 出境 车站 市场 服务 隐患 占比

Topic #16:

政策 退票 航班 公司 机票 请问 接待 疫情 规定 航空 电话 客服 北京 酒店 符合 全额 退款 申请 来访 上海 时间 高铁 民政部 东航 国航 南航 满足 广州 期间 手续费 本人 乘客 住房 深圳 去年 无法 方式 条件 往返 成都 杭州 宾馆 暂时 网络 西安 收取 减免 决策 非自愿 退票费

Topic #17:

顺义 复课 重新 不复 巡视员 四点 朝阳 北京 海淀 评估 响应 教委 措施 检测 二级 应该 情况 常态 严密 西城 丰台区 出现 人员 疫情 恢复 核酸 封闭 做到 大规模 基本 防疫 保障 报道 环境 继续 阳性 设计 从业 看到 央媒 停课 长期 教育 生活 安全 足够 这种 市场 持续 中国

Topic #18:

期望 结婚 材料 失望 半夜 宿舍 不对 娱乐 现在 封闭 耽误 疫情 办公室 工作 推迟 管理 连续 不要 心态 还要 唯一 病例 机会 进行 休息 整个 丰台区 人员 联赛 检测 社区 肺炎 努力 回家 小时 知道 开学 村口 时间 发热 心情 没有 开展 感觉 南门 公园 正在 医院 单位 核酸

Topic #19:

镇守 出入 背后 允许 祖国 农产品 新发地 武警 恐慌 加油 批发 原因 市场 北京 暂时 强大 临时 封闭 疫情 视频 不要 微博 水果 妈妈 民族 据说 露天 中国 蔬菜 一点点 室内 危险 一定 严重 真的 超市 希望 优势 每户 里面 西城 辞职 比对 地位 春天 直观 房子 相继 年末 让出

Topic #20:

市场 新发地 踏上 北京 知道 最后 青岛 批发 供应 疫情 周边 蔬菜 封路 必需品 双向 确诊 生活 封闭 病例 停运 保障 配送 大家 已经 总量 端午 蔓延 保护 去年 中国 欧洲 俄罗斯 改变 才能 东西 口罩 今年 提醒 退票 北京市 出门 做好 洗手 计划 农贸 力度 病毒 产品 应急 临时

Topic #21:

三文鱼 疫情 交易 病例 工作 进行 北京 新发地 市场 大厅 不要 肺炎 检测 冷冻 进口 患者 可能 单位 购买 新冠 心里 出现 封闭 丰台区 加拿大 牛羊肉 相关 运输 落实 人员 食品 价格 确诊 进去 以后 路过 打车 生鲜 核酸 超市 附近 已经 早上 开展 中国 日常 排队 避免 内容 被动

Topic #22:

措施 常态 情况 北京 疫情 保障 防疫 继续 传播 时刻 已经 程度 做到 临时 严密 应该 足够 影响 这种 现在 精细化 准备 设计 停摆 新发地 生活 安全 大规模 长期 经济 训练 一定 采用 持续 球队 接受 出来 中国 人民政府 比赛 开赛 企业 转化 好的 进入 做好 封路 开到 疫苗 变为

Topic #23:

病毒 疫情 新冠 感染 病例 传播 中国 市场 可能 海鲜 时间 确诊 控制 经济 措施 美国 出现 没有 发现 新增 印度 三文鱼 已经 有效 问题 专家 这次 应该 目前 数据 累计 全国 及时 爆发 污染 环境 发生 扩散 采取 情况 国家 英国 人数 进口 未来 抗疫 再次 增加 来看 发病

Topic #24:

学校 孩子 运动 毕业 课程 梦想 暑假 足球 美国 物品 开学 儿子 辅导 之前 教室 带走 家长们 疫情 要求 昨天 论文 出发 通知 眼前 悄悄 cm 答辩 暴乱 出门 这是 原因 第一 封城 接到 骚乱 憨爸 part 惊呆 遗留 最近 绝不 香港 暂停 器材 顺便 不说 推出 音响 姿势 品牌

Topic #25:

加油 训练 封闭 中国 一起 集训 即将 时光 看到 强大 基地 羡慕 球员 欢乐 明年 容易 乒乓球队 知道 奥运会 球场 特别 想到 战友 当年 站上 奥运 趣味 冠军们 赛场 乒乓球 新浪 国乒 比赛 进行 疫情 球队 nba 备战 复赛 计划 国安 热身赛 香河 集会 嘉伦 报道 开赛 联赛 俱乐部 中超

Topic #26:

生产 安全 需求 疫苗 抗体 全球 生物 产生 新冠 休假 目前 二手房 冠灭 中间 土豆 尝试 日均 车间 释放 中国 接种 中和 建成 数量 方面 看房 租房 阳转率 回应 江苏 中小学生 处于 特大 识别 购房者 开发商 售卖 率先 受试者 房东 哄抬 十四 整体 网签 毕业季 赔偿 星期四 高生物 gmp 人脸

Topic #27:

大厦 采购 西城区 时隔 房租 将近 伙食 到岗率 饭菜 减免 抵抗力 研究院 适用 预计 炸锅 连锁 隶属 超过 适当 下调 顺便 到岗 头脑 食材 物美 对抗 农贸市场 分别 转移 必需品 公司 限度 规定 新鲜 不住 导致 本地 有力 保洁 协调 个人 生活 整个 总是 状况 一下 疫情 同样 应急 直观

Topic #28:

城市 影响 下降 项目 环比 上周 北京 成交量 程序 成交 租金 受到 租赁 服务 出现 短期 面积 国内 新房 重点 四川 推动 继续 方面 二手房 交易量 上海 疫情 均价 备案 客户量 小幅 住宅 共有 分级 不同 房源量 下滑 平方米 应用 全覆盖 在线 报道 产权 相比 幅度 用于 消除 怀柔区 连续

Topic #29:

浙江 青岛 随之 福建 封闭 小区 较为 生活 山东 病毒 报告 想想 携带 安排 疫情 确诊 现在 措施 丰台区 志愿者 代购 保障 北京 最近 境外 需要 入境 吃饭 感染者 容易 风险 病人 病例 特殊 症状 干部 增加 专门 国际 居民 针对 严峻 新冠 组织 安心 环境 推送 购物 必要 介绍

### Resurgence – test-trace-isolate

Topic #0:

日本 韩国 泰国 酒吧 ⅲ期 冯子健 犯罪 埃及 第5 嫌疑人 时尚 合格 诊数 旅馆 卫生 kcdc 疑团 社会主义 总局 交换 战战 挨人 游泳 安检 打扫 一丝丝 满洲里 封尽封 取保 候审 名片 再生 推诿 前台 垃圾堆 国药 良方 白岩松 保驾护航 本意 国贸 重重 做客 搭乘 游泳池 布满 征兆 来人 查出 拥堵

Topic #1:

新冠 美国 病毒 中国 疫情 国家 控制 经济 全球 世界 社会 死亡 感染 印度 肺炎 可能 口罩 政府 人数 组织 人类 时间 问题 巴西 超过 危机 国际 表示 发展 能力 无法 特朗普 认为 保持 传播 健康 增加 数据 正在 国内 抗疫 总统 团队 这是 社交 需要 有效 人民 第一 大学

Topic #2:

球员 比赛 自我 球队 阳性 接受 联赛 训练 赛事 德约科维奇 俱乐部 表演赛 带领 维护 英超 中超 遭遇 排名 共有 尾声 盼望 外援 热刺 确认 资格 开赛 球迷 克罗地亚 对手 医学生 贝尔格莱德 第八 赛季 公布 职工 参赛 预告 基奇 主帅 落后 赢得 收集 西班牙人 细节 规则 小德 巡回赛 证实 攻坚 观看

Topic #3:

疫情 北京 控制 希望 隔离 真的 加油 生活 没有 大家 已经 今天 时间 现在 一定 这次 得到 觉得 过去 知道 感觉 很多 爆发 起来 相信 看到 不要 结束 工作 其实 早日 每天 在家 严重 孩子 尽快 很快 今年 正常 好的 朋友 本来 不是 出来 最后 期间 突然 最近 努力 第一

Topic #4:

成交 均价 客户量 哈萨克斯坦 纳扎尔巴耶夫 看房 稳中有降 队长 售楼处 业内人士 开发商 降幅 认购 郑重 房客 链家 首任 动力 以内 秘书 备案 营销 打消 购房者 毕业季 发文 顾虑 优惠 消退 激发 交房 买卖 廊坊 排序 上月 广场舞 预见 租房 卫星 延迟 排斥 不幸 官宣 广州市 商报 怀柔 共有 济南 房地产 合肥

Topic #5:

北京 隔离 疫情 政策 回来 需要 证明 退票 回去 现在 上海 机场 机票 回家 计划 出差 不是 取消 飞机 航班 知道 公司 出京 外地 准备 客服 期间 毕业生 收拾 当地 航空 考试 还要 出去 行李 自愿 端午 预约 出发 没有 考生 出行 已经 大概 高风险 落地 申请 杭州 十四 清零

Topic #6:

实际上 他家 搞定 慈善 船员 哥伦比亚 职责 wh 县城 品类 傲慢 面膜 中下旬 过来人 无效 步骤 护肤 香皂 昼夜 神圣 紧锣密鼓 年年 此人 拜访 招牌 这下子 头天 卖场 尾号 尊有 悲欢离合 麦德林 购物日 服务队 上百 披星戴月 厨房 大龄 传出 一并 事迹 某人 信誉 静心 创下 紧要 倾向于 十一 海产 测温枪

Topic #7:

短信 线上 企业 更新 app 休假 外国 正式 强制 零食 合同 持续 关注 工资 爱情 不通 地址 稳住 姨妈 心心 血糖 菲律宾 之际 发放 农业 上半年 拉升 开开 率先 肚子 下周一 变色 每月 考试 发送 交卷 无限 三大 会计 运营商 跳水 力量 问题 公平 不要脸 版本 sz 做梦 传达 恶心

Topic #8:

花钱 孤独 次方 孤岛 哲学 胡思乱想 醒醒 失眠 碰上 哈佛 带货 被子 承载 迷迷糊糊 悲催 寄托 半夜 作祟 结构化 真题 班长 裹挟 同城 萎靡 一波三折 启发 坏人 独处 主观 演讲 中英文 谈谈 商务部 部委 商务局 初心 信誉 百态 弱势 性格 追问 快捷 手记 现状 舍得 日头 关在 县长 咣当 偷偷

Topic #9:

隔离带 机动车 主路 滞留 行驶 领取 路程 拉起 申诉 二四六 驳回 大红门街道 疲劳 一阵子 商城 遮阳棚 人情味 肉类 马路 可否 学车 违法 更改 停车场 处罚 快捷 南里 膝盖 不许 打工 限行 想像 行人 挑战 执行 随后 做咽 三点 吓人 露天 拒接 违反 路线 咋办 无奈 狂风 清楚 尾号 有担当 适应

Topic #10:

安全 保护 到底 禁止 保证 粉丝 安排 合理 尊重 艺人 明确 无法 the 助理 各家 听一博 笑话 安保 饮食 全区 睡眠 接机 诉求 路人 基本 后援 大区 眼里 官博 途中 石家庄 佛罗里达 计算 录音 卫生局 好虞书欣 走vip 加热 去测 相邻 康码 良心 报送 教养 民事 黄村镇 扫健 疲劳 血压 遛弯儿

Topic #11:

酒店 返回 新西兰 前往 离开 司机 河北 妻子 就餐 到达 隔离 出租车 卫生部 安新县 摊位 女儿 抵达 内皮尔 轨迹 奥克兰 步行 自驾 大学 复诊 自驾车 近距离 散步 理工 保定市 丈夫 客人 拍摄 女性 端村镇 期间 设施 夫妇 乐园 安新 上个月 附属 父母 公众 惠灵顿 一同 冯某 王某 这几年 对面 驾车

Topic #12:

学校 学生 老师 开学 宿舍 女孩 上学 考场 强制 赶上 上课 今年 家长 男孩 请问 天后 要求 疫情 规定 现在 放假 大学 东西 物品 相关 空调 大厦 国家 执行 工作 开会 校园 积极 邮寄 安排 银行 房租 统一 网球 顺丰 工地 整理 延期 严格 学院 傍晚 防控 开放 保洁 树立

Topic #13:

冲击 施工 租赁 压路机 工程 顺四条 厂家 房源量 碾压 回补 湖人 休闲 餐员 乡红寺村 梅花碾 适度 周度 城带 古典 动力 稳中有降 以内 备案 强化 客户量 创新 指导 科技 服务 迎来 下发 gdp 优鲜 好的 廊坊 高峰 综合 挣钱 收益 老胡 提供 下一代 水果摊 季后赛 出自 科学 侧面 果多 广州市 大局

Topic #14:

参加 代表 解除 兄弟 可惜 娱乐 隔离 王一博 现场 心意 医疗队 礼物 天天 湖南 疫情 表达 承包 大家 援鄂 微博 视频 uniq wy 答谢宴 懒政 南山 入场 大峡谷 金属 专列 启程 胶囊 堪忧 娱文 l一天 分分钟 全货机 o37182 达卡 长沙站 逝世 上装 田地 邯郸市 高价 衣物 记错 不难 四点钟 主唱

Topic #15:

市场 非常 轨道 上涨 继续 这种 表现 个股 操作 趋势 明显 指数 热点 形成 经济 一定 集体 运行 反弹 格局 赚钱 特征 难度 人气 短线 控制 补涨 股指 疫情 区间 科技 仓位 比较 震荡 大幅 维持 为主 上升 目前 冲击 本周 方向 没有 保持 不错 业绩 属于 现在 节奏 影响

Topic #16:

检测 隔离 核酸 病例 确诊 北京 阴性 新增 地区 需要 集中 报告 居家 密切 进行 观察 症状 风险 累计 感染者 目前 人员 新发地 接触者 接触 要求 阳性 新冠 肺炎 输入 境外 医学 医院 自费 现在 北京市 全部 疑似 本地 截至 来自 强制 请问 骑手 高风险 河北 健康 规定 死亡 河北省

Topic #17:

发生率 假阴性 是非 编制 过早 大师 五分之一 建房 中房 租赁贷 鼻涕 违规 充当 讲讲 有毒 租客 展开 仓促 咽部 待检 退租 双管齐下 变相 试剂 一日 灰暗 美帝 零号 典型 有时 好不 不等 名称 三甲 冠肺 内科 国安 味觉 五六 嗅觉 众人 缘由 出自 约翰霍普金斯 一时 通常 轻度 误差 不明 后浪

Topic #18:

视频 微博 隔离 期间 法国 一起 运动 舞蹈 音乐 百事 百事可乐 陪伴 总监 午餐 薯片 警察 第四 企宣 志敏 事务部 早餐 乐事 过程 创作 公益 森林 穿上 表演 在家 第128 暖心 老王 干活 天空 镜头 还给 劳动力 舞者 艺术 入境 使用 分厂 下载 成年人 共同 体重 回家 cp 去世 顺利

Topic #19:

龙虾 大风 天佑 天伦锦城 天亮 天下 天上 天一 大龄 大面积 天信 大雨 大院 大陆 大队 大门口 大门 大量 天使 天儿 大方向 天数 天猫 天然 天灾 天津市 天津 天气 天时 天才 天后 天天 天大 天堂 天坛 天地 天命 天呐 大酒店 大都市 大都 大洋路 大爷 大热天 大火 大涨 大海 大浪淘沙 大浪 大法

Topic #20:

天津 省级 绿色 不用 轻型 甘肃 高铁 啤酒 面包 美食 汉族 京津冀 堂食 米饭 蛋糕 回族 欢乐 认证 口味 角色 吃到 搭配 教师 六月 甘肃省 外媒 安利 养生 雅得 mu7792 转为 南昌 油条 看上去 节日 犯法 骑行 分离 特利 自沙 收回 果果 江西 樱花 战战兢兢 少女 泰山 噩耗 当今 随地

Topic #21:

患者 治疗 症状 发热 咳嗽 糖尿病 独自 乏力 温岭市 咽痛 胸闷 发动 间断 肿瘤 予以 嘉兴市 头晕 年龄 激素 畏寒 高热 离点 警觉 指隔 糖皮质 系统化 头痛 窘迫 咳痰 京史 温岭 一万 大水 漫灌 水煮 l王轩 台州市 吴某某 心头 常理 浙江省 南湖区 诸君 休克 少痰 少部分 肾功能 重型 接报 有点儿

Topic #22:

评论 这位 小伙 吉林 拜托 勇敢 转发 讲述 病区 变身 误入 吴尊友 医者 守护人 展示 这家 兴趣 花费 卫视 报道 吴尊 违反 宵禁 面罩 o吴 已经 食客 引进 交出 新冠性 配图 归根结底 邓伦 同意 看错 加试 答卷 米兰 沙特阿拉伯 总计 卧槽 西藏 旅行史 dl 大屏 为重 小妹 鸡汤 出镜 首善

Topic #23:

疫情 北京 控制 病毒 市场 病例 出现 新冠 已经 新发地 防控 感染 措施 目前 确诊 可能 传播 专家 新增 发现 时间 采取 中国 武汉 海鲜 情况 没有 肺炎 减少 范围 相关 批发 全国 进行 表示 影响 这次 中心 得到 排查 城市 发生 及时 有效 应该 三文鱼 严格 扩散 疾控 食品

Topic #24:

电子 肖战 鲍威尔 有所 停靠 趋于 主席 平等 听证会 相见 长城 重申 地段 运输船 参议院 釜山港 开场 上映 偏向 估值 脚跟 站稳 xz x玖 daytoy 少年团 性价比 永久性 片儿 作证 错觉 相册 产出 l凤凰 容身 白中 参议员 族裔 上百 衰退 失业 倒闭 传出 四合院 就业 顺遂 提到 年度 有种 践行

Topic #25:

德国 英国 啊啊 游客 持有 连续 爆发 历史 隔离 肉联厂 西班牙 抗议 石油 继续 解封 庆祝 民众 toennies 预计 租金 封锁 事件 礼拜 化工 亚洲 权利 北威州 晚间 第六 浙江 旅游业 房价 川普 州长 美国 三年 沙特 油罐车 局面 加工厂 大厂 甲醇 柏林 几十万 丹麦 示威 新低 化工品 几月 伊拉克

Topic #26:

免疫力 列车 认知 明春 核实 机体 财务 道德 体质 向上 交际 课堂 人性 立场 空中 怨天尤人 勇往直前 天灾 价值观 拮据 混混沌沌 仇视 良知 大浪淘沙 麻将 阅历 扑克 死灰复燃 第31 航城 生肉 廊坊市 懈怠 赤裸裸 谊品 深思 惨痛 永定 o钟 下水 杀灭 到家 必须品 飞沫 勇气 艺术园 来广营 灵魂 复课 自律

Topic #27:

检测 隔离 新发地 北京 没有 核酸 小区 现在 今天 口罩 疫情 居家 不是 通知 已经 大家 社区 知道 排查 政府 昨天 电话 上班 在家 出来 很多 问题 公司 工作 同事 有人 一下 不要 单位 风险 情况 应该 这是 要求 路过 防疫 地方 时间 强制 配合 可能 大数据 需要 小时 出门

Topic #28:

人员 工作 社区 排查 检测 进行 北京 医院 防控 新发地 市场 核酸 疫情 小区 北京市 居民 管理 辛苦 医护 中心 做好 街道 开展 健康 所有 相关 要求 落实 单位 措施 周边 严格 医务 全面 保障 登记 防疫 重点 进入 机构 环境 发布会 情况 采样 封闭 疾控 安全 检查 配合 肺炎

Topic #29:

电影 观众 嗨皮 完赛 配带 意念 氧气瓶 认可 秀秀 美图 电视剧 安安静静 笑笑 光芒 这部 冰棍 演技 反对者 发肖战 上色 扣锅 手绘 全貌 卖惨 污水 跨国 飞到 夜店 声明 净化 麻痹 注射 好听 天上 飞扬 超话 发博 洛杉矶 现场 立刻 家中 学者 三重 得知 这不 拒绝 恶意 片子 疫苗 甩锅

### Resurgence – suspension of gathering

Topic #0:

增长 招聘 周四 季度 同比 进步 届生 就业 瑜伽课 单独 支撑 大柳树 小姑娘 周二 二月份 登陆 还好 完整 意大利 才华 心思 指标 下跌 动作 一季度 青春 奋斗 网球 去年 积极 正是 大量 台风 明天 家里蹲 老实 研发 北京 毕业生 算是 预定 疫情 留学 困难 行程 高校 取消 申请 定位 增加

Topic #1:

退票 退款 延长 购票 一边 携程 原因 疫情 付款 不通 受理 不予 维权 排面 优讯 催促 网上 理会 时长 天下 生产 澄秀园 天秀花园 懒惰 键盘 常态 指望 恨不得 屏幕 回复 等待 消息 少有 邻国 散步 大伙 雅思 离开 考试 减免 了解 超过 上课 真是 重回 电话 时间 不要 暂停 估计

Topic #2:

br 快递 发货 升级 电商 跨境 疫情 营养 区域 北京 语言 少数 停止 更新 监管 截止 瑟瑟 发抖 游客 莫名其妙 抵抗 商务部 宝宝 添加 尊敬 疾病 完善 丰富 一面 合理 门口 原则 办事 当事人 执法 新华社 中级 方案 ceo 空白 四六级 不怎么 要求 良好 频率 时期 变化 寒假 申请 汇英

Topic #3:

短租 演出 门雍 找找 崇文 南次 勿扰 蒲黄榆 东单 直租 宫大望路 居中 时间 原来 房东 推迟 付款 拖延症 直接 明年 解禁 今日 今晚 疫情 之后 入住 室友 二环 电梯 哥哥 小伙伴 加上 发表 方便 哥伦比亚 放心 地铁 吃喝 格拉斯哥 过上 缘由 不幸 工作日 不够 盛会 石景山区 保险 陆续 点击 傍晚

Topic #4:

疫情 防控 北京市 要求 各类 暂停 体育 工作 活动 时间 赛事 安全 落实 安排 形势 自即日 举办 日元 风险 带来 减少 全市 保障 切实 身体 生命 人员 另行 人民 变化 流动 恢复 聚集 健康 通知 期间 开放 北京 相关 检测 新冠 学生 单位 停止 消息 影响 上午 执行 严格 安心

Topic #5:

批发 市场 东寺渠 北京 农副产品 暂停 一口气 工作 计划 推迟 结婚 烦躁 上来 营业 青岛 病毒 平谷 记者 防控 抽检 冠状 山东 平谷区 新型 病例 消息 相关 获悉 食品 疫情 目前 到访 肺炎 刚刚 进行 青年报 需要 新增 托福 刺猬 再三 患者 必然 位数 控告信 网络 原有 隐私乐 义务 劳资

Topic #6:

复赛 球队 奥运会 球员 cba 东京 运动 nba 奥运 提前 联盟 英超 男篮 运动员 支持 取消 奥林匹克日 滑手 健儿 奥组委 参赛 滑板 赛季 飞往 破门 种族 活跃 女篮 燃炸 呐喊 解散 早报 德甲 武磊 房价 奥林匹克 奋斗 女足 改到 项目 mvp 奥兰多 奥委会 西部 官员 skate 积分赛 赚取 认可 world

Topic #7:

印度 数据 缩短 公布 单日 智利 国家 行为 措施 架次 疫情 严厉 灾难 全国 非洲 we 宣布 停止 vip this 无效 请求 but 封锁 宵禁 中国 穿着 拿出 收益 良心 良退 收割 累计 历史 防护服 诱导 what 落实 增幅 are 截至 刷新 u17 世青赛 监管 停工 打击 尤其 延长 北区

Topic #8:

疫情 取消 北京 今天 真的 计划 现在 没有 本来 知道 希望 延期 突然 晚上 原因 推迟 机票 已经 觉得 通知 回家 不是 一下 今年 公司 朋友 最后 在家 明天 行程 只能 结束 真是 隔离 生活 一起 感觉 起来 端午 上班 航班 出去 家里 工作 酒店 有点 收到 喜欢 暂停 最近

Topic #9:

tulsa室 terry 晓峰 外秀 玻璃 感染者 增加 保加利亚 紧急 状态 该国 延长 决定 国家 不断 数量 肺炎 消息 防弹 宣布 新冠 目前 总理 见识 基层 传说 骑车 死者 毕业 六大 运行 居留 能否 不予 单元 清零 来看 答复 出去 结课 退房 接到 罚款 哈哈哈 来来回回 限定 意见 偶像 划算 广外

Topic #10:

存在 心理 心理学 意识 读心 迷信 社会 主持 内心 访谈 参与者 法官 思想 情绪 合理 人类 脱口秀 鼓吹 一起 依然 活动 精神 西方 没有 相互 情感 大神 悖论 罪犯 不是 认同 造成 思维 主持人 更加 学者 蔓延 精英 认可 可怕 逻辑 治疗 直到 来自 别人 几百 央视 火爆 频道 世界

Topic #11:

疫情 防控 北京 暂停 工作 北京市 通知 恢复 体育 活动 要求 运营 措施 时间 做好 停止 人员 响应 机场 二级 另行 场所 年级 安全 相关 管理 落实 健康 肺炎 发布 开放 形势 举办 聚集 班线 赛事 业务 交通 一律 严格 减少 保障 中小学 带来 全市 安排 廊坊 出行 单位 各项

Topic #12:

停止 人民 疫情 世界 地球 脚步 暂时 被迫 一线 每个 跑步 看到 翻译 邻居 都会 两点 带领 普通人 大海 破坏 海洋 伤害 室内 疫病 时间 go 日子 保护 旅行 只得 各国 立刻 始终 转变 毁灭 稿子 对付 欧洲杯 意思 承受 体验 为时晚矣 早醒 领导人 此次 视为 李子柒 登顶 熟悉 唯有

Topic #13:

底层 兼顾 本当 性价比 考试 认同 本质 优秀 改期 终于 安排 人命 英语 期待 家庭 比较 还要 这次 经历 学校 学习 第一 工作 取消 疫情 好运 穿过 生存 听听 计算 中超 中都 锐减 终端 轻松 整个 周岁 对抗性 高速 卫国 股东 好说 商务 股市 帐篷 门诊 来园 内蒙古 遭遇 火锅

Topic #14:

生怕 翻身 面基 格外 三口 毛病 母亲 发出 小伙伴 折腾 多久 今晚 醒来 疫情 踏实 越来越 每次 线下 学习 一起 严重 起来 明天 回来 活动 在家 现在 希望 今天 取消 安排 总裁 课程 集团 相信 都会 思路 供应链 认知 天津 时间 能够 成功 课堂 培训 选择 受到 影响 乐队 速度

Topic #15:

疫情 影响 推迟 新冠 今年 宣布 举行 美国 延期 原定 英国 时间 取消 进行 电影 可能 举办 计划 表示 颁奖 上映 全球 明年 典礼 奥斯卡 政府 报道 活动 已经 中国 目前 决定 经济 国内 国家 日本 世界 日期 肺炎 原因 第一 国际 行业 艺术 延长 德国 此前 考虑 公司 措施

Topic #16:

视频 微博 延期 签证 演唱会 更新 游戏 无限 播出 移民 会员 线上 外国人 女孩 粉丝 分钟 改为 发布 现在 冒险 拉夫堡 少女 入境 漫展 行政令 脱颖而出 许可 公告 签署 海绵 半泽直树 今天 外交部 定档 电影院 周边 睡眠 h1b 出道 转卡 体验 居留 封国 传媒 o6月 届时 大门 点赞 事儿 第二三

Topic #17:

个头 低年级 小学生 好不 定于 难受 开学 下周一 悲伤 中国 澳大利亚 病毒 刚刚 发现 延期 新增 今天 不是 开心 病例 经济 正在 设施 产品 实际上 需要 进口 价格 能源 关系 并未 增加 要求 基础 程度 美元 第一 早已 限制 导致 国际 调查 年初 采取 近期 使用 依赖 产能 上个月 关税

Topic #18:

毕业 特朗普 典礼 论文 向前 大学生 明明 实验 相约 毕业季 格外 毕业云 答辩 开题 携手 暖心 寄语 智云 道别 圆梦 转眼 警察 书记 进度 反对 感动 感慨 高层 博文 该死 遥远 无意 大都 顺便 职责 大姨妈 冲突 匆匆忙忙 运动 难忘 不说 表达 无情 其他人 最近 半年 短裤 取消 疫情 时间

Topic #19:

放学 exo live 公演 演出 随机 校方 夏季 but 老人 孩子 消息 一万 中午 盼望 据悉 娱乐 疫情 马路 明星 很多 加盟 被迫 温度 参加 肺炎 延期 出门 原定 举办 影响 新冠 小时 统一 心里 感染 高温 家长 开学 只能 今天 安排 知道 下午 学生 以上 家里 这是 推迟 生肉

Topic #20:

疫情 北京 取消 时间 没有 大家 现在 已经 地区 学校 工作 影响 希望 不是 情况 学生 问题 很多 期间 政策 可能 需要 小区 所有 推迟 风险 严重 生活 航班 今天 不要 预约 安全 目前 口罩 老师 停止 要求 再次 恢复 爆发 这种 应该 安排 一定 继续 非常 学习 新闻 控制

Topic #21:

考试 高考 面试 成绩 考生 复习 高三 会计 高中 笔试 考场 知识 初级 学子 科目 平凡 命运 认真 初三 高考生 孩子们 自律 延迟 注定 财政部 考出 冲刺 晚安 重点 听课 财管 笑话 心仪 大好 全科 拼搏 一眼 如约而至 步伐 非典 奔赴 拉开 资产 全力以赴 出版 难度 倒是 大批 接近 会否

Topic #22:

意识 人类 迷信 社会 进化 恐惧 纠缠 读心 情绪 臆想 时代 方法 鼓吹 访谈 存在 内心 学会 前行 不断 越来越 认识 网络 心理学 思想 心理 验证 大脑 相互 工业 顽疾 沟通 代替 客观 主持 漩涡 侥幸 造成 文明 革命 先烈 面对 虚拟 无法 几千 胡思乱想 危害 焦虑 阻碍 空间 家长

Topic #23:

比赛 联赛 时间 赛季 没有 疫情 中超 重启 火车 推迟 需要 正式 中介 入住 开赛 北京 赛事 中级 位置 地铁 短租 积分 西甲 方案 小伙伴 缅甸 班次 意甲 重赛 电梯 谢谢 伙伴 赛程 下赛季 已经 室友 毕竟 本人 崇文 二环 方便 沙特 疏解 压缩 蒲黄榆 直租 勿扰 居中 宫大望路 门雍

Topic #24:

本身 多次 道德 老婆 佩服 北戴河 推荐 电影 兄弟 一家人 取消 视频 孩子 天天 不行 好好 三亚 新冠 晚上 后来 病例 节奏 微博 已经 应该 周围 有点 半年 北京 机票 历史 昨天 新增 科学 来不及 疫情 地方 突然 健身房 预约 网站 上映 其实 我国 一辈子 有效 表示 说法 变成 细节

Topic #25:

公园 牙齿 推荐 近期 靠谱 觉得 重要 对比 无聊 在家 关心 地方 难受 牙套 这次 自卑 矫正 矫正牙齿 姐妹们 郑州 虎牙 嘴巴 畸形 金属 阻止 舌侧 自锁 没法 钢丝 平直 覆合 面型 下巴 颏沟 传统 石景山区 两边 下意识 折腾 数字化 肌肉 笑容 疼痛 中等 世纪 颠覆 帝都 天才 不久 往往

Topic #26:

病例 确诊 北京 医院 检测 新增 患者 新冠 出现 肺炎 接触 北京市 阳性 密切 新发地 大爷 停止 累计 丰台区 西城 暂停 门诊 街道 症状 报告 巴西 以来 人员 死亡 核酸 地坛 所有 住院 战时 感染者 关注 集中 发病 连续 管控 中心 隔离 美国 西城区 机构 返回 急诊 生活 本地 发热

Topic #27:

检测 核酸 市场 北京 新发地 人员 暂停 进行 营业 批发 社区 目前 隔离 所有 外卖 通知 下午 高风险 骑手 部分 海鲜 丰台 相关 记者 区域 全部 已经 确诊 今天 商户 快递 关闭 病例 接单 全面 丰台区 排查 观察 回应 大厅 交易 部门 检查 花乡 昨天 工作 消息 表示 上午 封闭

Topic #28:

无所谓 促进 医学生 常态化 消费 其实 已经 经济 延期 口罩 奖金 科室 救人 十万火急 开拍 青少年 感染科 人话 医务 部长 医师 致敬 帮帮 潜伏期 战场 北青报 挨家挨户 干货 自我 警察 关税 诱惑 仿佛 ct 感慨 值钱 希腊 上周末 现状 风景线 表现 过期 运送 下旬 首映 五分之一 外贸 机器 频繁 嫌疑

Topic #29:

病毒 新冠 进口 感染 市场 新发地 三文鱼 暂停 美国 海鲜 产品 停止 中国 食品 企业 北京 可能 武汉 检测 蔬菜 传播 肉类 公司 员工 国家 超市 供应 冠状 海关 发现 已经 海外 发生 国内 人数 聚集性 毒株 生鲜 问题 猪肉 生产 批发 延长 是否 疫情 动物 污染 情况 接触 运输
